# Recent Advances in Biological Recycling of Polyethylene Terephthalate (PET) Plastic Wastes

**DOI:** 10.3390/bioengineering9030098

**Published:** 2022-02-27

**Authors:** Ya-Hue Valerie Soong, Margaret J. Sobkowicz, Dongming Xie

**Affiliations:** 1 Department of Chemical Engineering, University of Massachusetts Lowell, Lowell, MA 01854, USA; yahuevalerie_soong@student.uml.edu; 2 Department of Plastics Engineering, University of Massachusetts Lowell, Lowell, MA 01854, USA; margaret_sobkowiczkline@uml.edu

**Keywords:** PET, plastic recycling, biodegradation, bioconversion, PET hydrolase, cutinase

## Abstract

Polyethylene terephthalate (PET) is one of the most commonly used polyester plastics worldwide but is extremely difficult to be hydrolyzed in a natural environment. PET plastic is an inexpensive, lightweight, and durable material, which can readily be molded into an assortment of products that are used in a broad range of applications. Most PET is used for single-use packaging materials, such as disposable consumer items and packaging. Although PET plastics are a valuable resource in many aspects, the proliferation of plastic products in the last several decades have resulted in a negative environmental footprint. The long-term risk of released PET waste in the environment poses a serious threat to ecosystems, food safety, and even human health in modern society. Recycling is one of the most important actions currently available to reduce these impacts. Current clean-up strategies have attempted to alleviate the adverse impacts of PET pollution but are unable to compete with the increasing quantities of PET waste exposed to the environment. In this review paper, current PET recycling methods to improve life cycle and waste management are discussed, which can be further implemented to reduce plastics pollution and its impacts on health and environment. Compared with conventional mechanical and chemical recycling processes, the biotechnological recycling of PET involves enzymatic degradation of the waste PET and the followed bioconversion of degraded PET monomers into value-added chemicals. This approach creates a circular PET economy by recycling waste PET or upcycling it into more valuable products with minimal environmental footprint.

## 1. Introduction

Plastics are composed of a broad spectrum of high molecular weight polymers derived from synthetic, semi-synthetic, or natural compounds, assembled in a repeating pattern [1,2]. Plastics can be readily molded into any shape and form by certain degree of polymerization and melting processing [3,4]. Being able to design or engineer polymers gives plastics incredibly versatility, with unique characteristics in strength, flexibility, durability, stress resistance, lightness, and electrical insulation.

**Figure 1:**
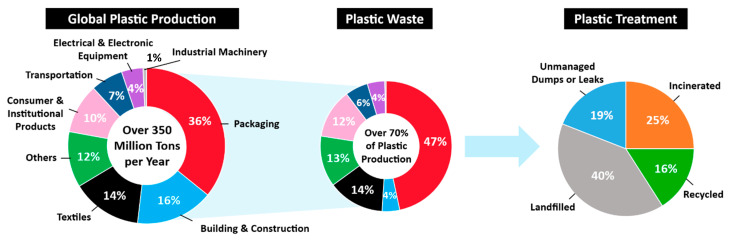
Global plastic production. The most plastic is destined for single use packaging. More than 240 million tons of plastic waste are generated every year. About 40% of plastic waste has accumulated in landfills and 25% has been incinerated [7,8].

More than 350 million tons of plastics are being produced worldwide annually in various applications, including packaging, building and construction, textile, consumer and institutional products, transportation, electrical and electronic equipment, and industrial machinery [5] (Figure 1). Although plastics are valuable resources in many aspects, the proliferation of plastic products in the last several decades have resulted in a negative environmental footprint due to poor recycling rates after their first use. Despite this obvious problem, plastic production volume is expected to continuously increase during the next a few decades [6]. Currently, about 70% of global plastics are found as waste. Only around 41% of post-consumer plastic waste is recovered by recycling and incineration with energy generation process, whereas 40% is disposed of in landfills and 19% ends up in the oceans or on coastlines [7].

Plastics are mainly made as synthetic polymers, and a small portion are made as naturally occurring biopolymers. Owing to their low cost, the ease of manufacture and their versatile properties, synthetic polymers are used for many different products. The majority of synthetic plastics, including polyethylene (PE), polypropylene (PP), polystyrene (PS), polycarbonate (PC), polyvinylchloride (PVC), and polyethylene terephthalate (PET), are derived from fossil hydrocarbons, a non-renewable resource. Due to the increasing demand for plastic products and the lack of efficient and economical ways of recycling the used plastics, there has been a growing concern for plastic pollution in the environment. The long-term risk of hazardous chemicals from the released plastic wastes in the environment poses a serious threat to ecological systems and health problems. In particular, the micron-sized particles degraded from waste plastics, called microplastics, have now become a major pollutant in the ocean that threatens hundreds of thousands of marine lives due to ingestion, entanglement, and smothering [9,10,11,12,13].

Among all plastics, PET is the most abundant polyester manufactured in the world and has been widely used for beverage bottles, packaging, clothing, and carpeting. At the same time, large quantities of PET have also been released into the environment during the process of its production, application, and disposal [14]. It is estimated that it takes hundreds of years to completely degrade PET plastics by microorganisms in the environment. Now, the accumulation of PET wastes is continuously increasing and starting to threaten ecosystems across the globe.

Increasing environmental awareness has inspired the search for novel technologies and other more environmentally friendly solutions, which not only deal with the increasing amount of plastic waste but also reduce the dependency on petroleum resources as the building blocks for manufacturing PET and other types of plastics. Mechanical recycling of PET into new packaging and clothing fibers is a low-cost and well-established approach, but it often downgrades the material properties [6]. Bioconversion of PET wastes into value-added chemical products may provide great benefits for both controlling the plastic pollution and creating new biomanufacturing resources [14,15].

This review aims to summarize the current major advances in recycling technologies for plastic wastes, particularly for biorecycling of PET. We hope it can help provide general guidance and directions for developing a more sustainable and economical solution towards a circular economy of PET in the future.

First, conventional approaches for recycling of PET and other plastics are discussed, which include landfilling, incineration for energy recovery, downgauging and reuse of packaging plastic materials, mechanical recycling, and chemical recycling. After that, the recent progresses in the biodegradation of PET waste are compared and analyzed, which covers the methods of both microbial degradation and enzymatic degradation. The advantages, new opportunities, and challenges for using biological recycling approaches towards a circular economy of new plastic industry are also discussed.

## 2. PET Properties and Applications

Polyethylene terephthalate (PET) is a condensation polymer synthesized by the polymerization of terephthalic acid (TPA) and ethylene glycol (EG), or by transesterification of dimethyl terephthalate (DMT) and EG [14,15,16]. Since it was first developed by DuPont in the middle of the 1940s, PET has become one of the most commonly manufactured thermoplastics. Depending on the intended application and desired properties, virgin PET is produced at different specifications by controlling the polymerization conditions [17,18]. PET is primarily used as textile fibers and then applied to the fabrication of polymer films. Later, PET has been extensively used in injection blow molding applications to produce durable crystal-clear bottles and jars [19,20]. Patented by engineer Nathaniel Wyeth in 1973, PET plastic bottles quickly gained market acceptance and have grown worldwide in popularity and versatility to become a leading choice as beverage containers.

The demand for PET packaging, most commonly food and beverage packaging materials, is expected to grow in the coming years as it is increasingly being used as a replacement for glass and metal containers. Compared with other plastic materials used for packaging, PET plastics are more durable, transparent, light-weighted, non-reactive, shatterproof, thermally stable, cost-effective, and with higher pressure resistance, mechanic strength, and better barrier properties (i.e., impermeability for liquids and gases) [21]. As PET is extremely resistant to hydrolytic and enzymatic degradation, it makes PET very hard to be decomposed and therefore the majority of PET polymers manufactured today will persist for a considerable time, at least decades and probably for centuries. As a consequence, substantial quantities of end-of-life PET plastics are accumulating in landfills, global oceans, and natural ecosystems, affecting mainland and aquatic life negatively [22,23,24,25]. 

## 3. Conventional Approaches for Recycling of PET and Other Plastics

PET is the most abundantly used polymer in the world. About 56 million tons of PET are produced worldwide annually, most of which are for single-use packaging material, such as disposable consumer items and packaging. After a short first use, a staggering 95% of plastic packaging material value is lost to the economy, with the depreciation reaching up to $80–120 billion annually [26]. As of 2016, the major share of PET resin market by end segments was dominated by PET bottles, accounting for 71% of global PET resin demand. Among bottle grade PET resin, bottled water has the highest demand by end-use segment, then carbonated soft drinks, followed by other drinks, of which concern a share of 26.3%, 26.1%, and 18.6%, respectively (Global PET Resin Market, 2016). 

In view of increasing concerns over environmental protection, resource conservation, and the development of recovery technology, recycling has become a basic premise in the supply chain of PET bottles [27]. Collection programs for plastic waste have been implemented across many countries since the 1970s. However, only about 25% of the PET bottles are recycled in the United States. PET plastic materials can be recycled in various ways and the ease of recycling varies among package design and product type. Therefore, the increasing trend of PET waste management strategies, including recycling and recovery, is one of the solutions to reduce energy and resource depletion, avoid harmful emissions and minimize quantities of mismanaged plastic waste reaching the environment [28].

To establish an integrated plastic waste management system, the main strategies are focused on the four R’s hierarchy, specified as reduce, reuse, recycle, recover, which lead to improvements in the life cycle of plastics [29,30]. Recycling is the process of using recovered material to manufacture a new product or to recover energy once a material enters the waste stream [6]. 

### 3.1. Landfilling

Landfilling is considered the least desirable approach for plastic waste disposal, but space and sites for landfills are becoming scarce. Moreover, beyond the concerns related to collection, transportation, and long-term risks of contamination of soils and groundwater by toxic additives, give rise to the persistent organic pollutants [31]. Indeed, from the aspect of sustainability, a major drawback for landfills is that most of the material resources used to produce the plastic are hard to recover, suggesting that the material flow is linear rather than cyclic [6]. Therefore, landfills should only be used as a last resort, to accommodate wastes resulting from recycling, feedstock production, or waste-to-energy [32]. 

### 3.2. Energy Recovery and Incineration

Energy recovery stands as the most resource-efficient solution available compared to landfills. Energy recovery from waste means the conversion of waste into usable heat, electricity, or fuel through a variety of processes, including incineration/combustion, gasification, and pyrolysis [33]. Incineration is the most commonly widespread thermal treatment, which is a process aimed at attaining the complete oxidation of all the suitable elemental species encompassed in the feedstock material [34]. 

Incineration of municipal solid waste (MSW) is a technology to treat waste while both exploiting the energy content of the material and reducing the amount of solid material to be landfilled. However, older incinerators are facing operational problems associated with the increase in plastic content in MSW. The reason for this is the high-yielding calorific value of plastics (often in excess of 40 MJ/kg) due to the high content of carbon and hydrogen [35]. Incineration of plastics raises a concern about the release of hazardous substances, such as the fly and bottom ash containing toxic residues (e.g., lead and cadmium), the leakage of toxic chemical compounds (e.g., dioxins, polychlorinated biphenyls, and furans), and the emission of greenhouse gases (e.g., carbon dioxide, methane, and nitrous oxide) [35,36,37,38]. In addition, incineration does not reduce the demand for new material.

Incineration can be used with recovery of some of the energy content in the plastic. For example, burning of plastics and other MSW can produce heat and steam to turn turbine blades and generate electricity for the local grid [39]. As many environmental regulations have been implemented for pursuing a more sustainable recycling-oriented society, incineration has been widely considered to be ecologically unacceptable in the last decade [40].

### 3.3. Downgauging and Reuse of Packaging Plastic Materials

Downgauging and reuse of plastic packaging are an economic imperative and the means to achieve sustainability. Downgauging allows packagers to offer the same products with higher product-to-package ratios by using thinner packaging materials, and thereby reduces the amount of material needed. Refilling and reusing plastic containers directly reduces the demand for disposable plastic. Studies have found that if glass and PET bottles were refilled and reused up to 25–35 times, overall beer and soft drink container waste would be reduced by 73.6% [41]. As compared to single-use throwaway plastic bottles, significant reduction in waste and energy consumption can be achieved with 7–8 reuses of a single bottle. 

PET is an inert plastic and has been safely used for many years. The toxicity study investigation on the use of PET for refillable bottles has exhibited that none of the toxic substances leached into its contents, either when a beverage is stored unopened, or when bottles are refilled or frozen. It indicates that a PET bottle itself poses no danger when refilled and could be considered as a practical candidate for refillable containers [42,43]. Moreover, PET has undergone rigorous testing under United States Food and Drug Administration (FDA) guidelines to ensure its safety as a food and beverage container suitable for storage and reuse. Refillable PET bottles lower the carbon footprint and save up to 40% of raw materials and 50% of greenhouse gas emissions [44]. The most robust refillable systems can be found in Latin American, where the refillable PET carbonated soft drink bottles have been used for many years with no safety issues. However, opened bottles can harbor bacteria, as will glasses or any other beverage containers. All drinking containers, including PET bottles, should be cleaned and washed thoroughly prior to reuse.

### 3.4. Four Categories of Plastic Recycling 

**Figure 2:**
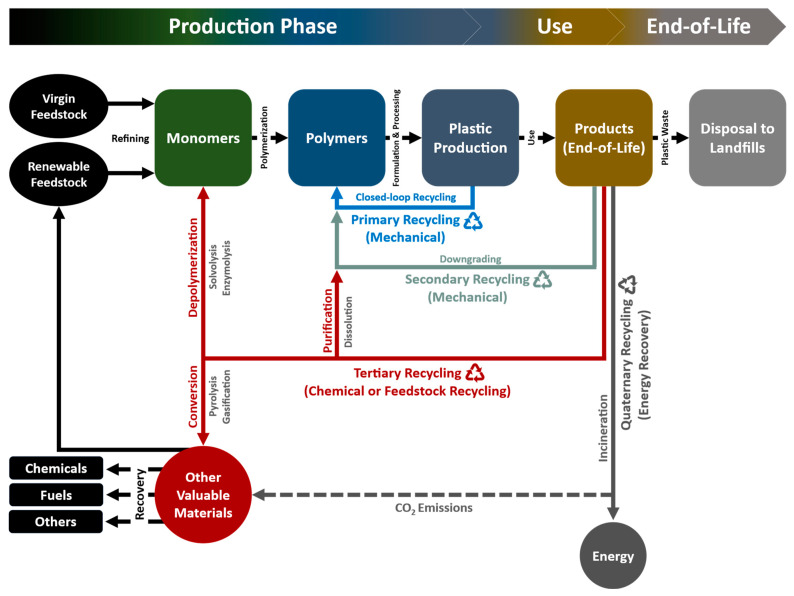
Plastic waste recycling and recovery routes [6,45,46].

As applied to plastic packaging, terminology for plastic recycling according to American Society for Testing and Materials (ASTM) D5033 definitions includes four categories: primary (mechanical reprocessing of scrap materials into products with equivalent properties), secondary (mechanical reprocessing of used materials into products requiring lower properties), tertiary (recovery of valuable chemical constituents, such as monomers or additives) and quaternary (recovery of energy) as presented in Figure 2 [6,45,46]. The transformation of the waste to more valuable materials or products via reutilization or recycling is called valorization. The primary recycling mainly covers the reuse of the waste plastics, while the tertiary and quaternary categories fall into the valorizing. 

#### 3.4.1. Primary Recycling

The primary recycling of plastics has been described as the process of reconversion of clean, uncontaminated, and single-type plastic waste into its original pellet or resin form, and reuse of materials for the very same applications [47]. The recovered plastics used in products have equivalent performance characteristics to those made using virgin plastics, which conserves the great amount of energy and cost. Ideally, primary recycling is often referred to as closed-loop recycling. However, closed-loop recycling is most practical only when the polymer constituent can be effectively separated from contamination sources and stabilized against degradation during reprocessing and subsequent use [6]. Owing to the purity and stability requirements, there is an obvious limitation on the number of cycles and narrow range of applications for each plastic waste [48]. Recovery of PET from scrap bottles used in the production of new bottles is an example of this recycling category.

#### 3.4.2. Secondary Recycling (Mechanical Recycling)

Secondary recycling works as downgrading converts scrap plastic or waste into products that have less demanding performance requirements than the original application via physical means. In general, the polymeric waste is reprocessed into granules by conventional extrusion after being separated from its associated contaminants. The main drawback of this mechanical recycling process is deterioration of the properties of the product, mainly due to the decrease in molecular weight after each cycle as a result of chain-scissions [49]. This approach often requires reformulation to meet specification of the new product. Practically, PET and polyethylenes, such as high-density polyethylene (HDPE), low-density polyethylene (LDPE), and linear low-density polyethylene (LLDPE), are able to be recycled by a mechanical process, while fluoropolymers are unable to be processed mechanically.

#### 3.4.3. Tertiary Recycling (Chemical or Feedstock Recycling)

Tertiary recycling is the process of decomposing waste plastic into their building blocks (i.e., monomers, oligomers, mixtures of the hydrocarbon compounds or other valuable low molecular weight fragments). The decomposed products can subsequently be used as feedstock for the production of the plastic materials or as fuels for the production of automotive, gasoline, jet fuel, and diesel products. Chemical recycling of PET has been more successful as depolymerization under milder conditions is possible. PET plastic can be broken down into dimethyl terephthalate and diols by glycolysis to make unsaturated polyester resins or to remanufacture virgin PET [50]. In spite of all its advantages, chemical recycling is not a promising process for economic sustainability mainly due to the energy costs [51].

#### 3.4.4. Quaternary Recycling (Energy Recovery)

High calorific value is one of the physical property of plastics that is quite valuable. Energy recovered from plastic waste can make a primary contribution to energy supply. Quaternary recycling refers to the energy recovery from plastic waste by incineration [8,52]. This is currently the most effective approach to reduce the volume of organic materials but retaining little value. Nonetheless, quaternary recycling is viewed as ecologically unacceptable by reason of considerable toxic substances in smoke and ashes.

**Figure 3:**
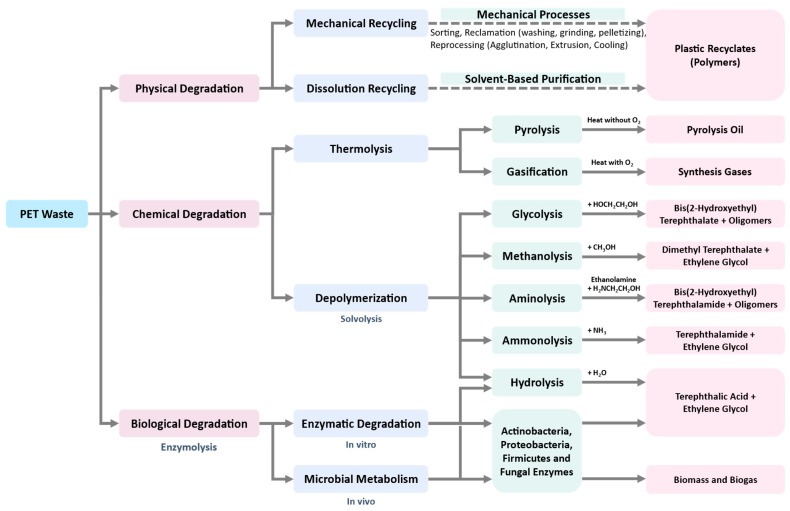
Physical, chemical, and biological approaches for PET recycling [2,54,55].

Most of the recycling techniques described above for PET wastes have not been applied at a large scale. Development of new recycling technologies is an urgent need in the present situation, which diminishes the consumption of energy, increases the amount or value of products, and reduces or completely eliminates toxic wastes in a sustainable, economically feasible and socially responsible manner. All physical, chemical, and biological approaches that can be used for PET recycling are summarized in Figure 3. Biological recycling is considered as one of the promising solutions, and has gained popularity in recent years, especially by using microbial or enzymatic degradation to produce downcycle feedstocks [53]. Furthermore, enzymatic technology could be applicable at a large scale for dealing with production, recycling, and detoxifying of plastic waste.

## 4. PET Recycling via Microbial Degradation

Biodegradation is defined as the decomposition or degradation of organic substances by the actions of biological entities, such as microorganisms (i.e., bacteria, fungi, and marine microalgae) or enzymes [56,57,58], which is considered to occur after or concomitant with abiotic degradation. Although synthetic polymers were once considered resistant to microbial degradation, more recent studies have demonstrated that certain microbes have evolved to produce a variety of hydrolytic enzymes that allow them to degrade and process polymers. 

**Figure 4:**
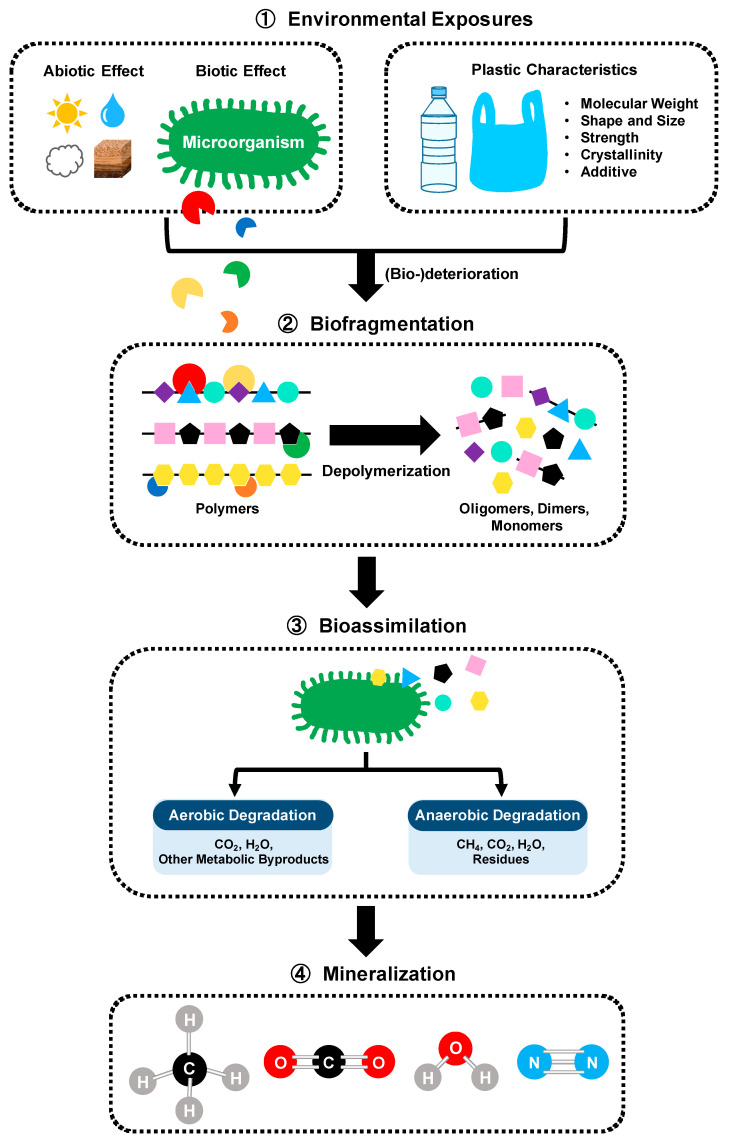
Mechanism of microbial degradation of plastics.

Biodegradation of polymer involving microorganisms can be performed by a microbial community or a single strain through several steps (Figure 4) [59,60]. First, the macrostructure of the plastic matrix is fragmented into small pieces due to abiotic and biotic factors (i.e., solar light, irradiation, oxygen, pH, moisture, temperature, pressure, and abrasion). Microorganisms capable of using plastics as a carbon source and energy attach to the polymers, followed by the surface colonization and biofilm formation [61,62]. Biodeterioration by biofilm communities growing on the surface and inside the plastics enlarges the pore size and facilitates the cracks. Biofragmentation relates to the action of extracellular polymer-degrading enzymes (i.e., oxygenases, ureases, esterases, lipases, proteases, depolymerases, cutinases, etc.) secreted from the microbial colonies. These enzymes enable lowering the molecular weight and shorting the carbon-chain backbone of polymers by total or partial depolymerization of polymers into oligomers, dimers and then monomers that can be assimilated by the cells. Bioassimilation refers to the integration of atoms inside the cells. Microorganisms can easily metabolize most small molecules (oligomers less than 600 Daltons) and convert the polymer carbon/nitrogen into building blocks of cells, which is contributing to the increasing of biomass. The ultimate step of the polymer biodegradation is the mineralization, which is the excretion of completed oxidized metabolites, such as CO_2_, CH_4_, N_2_, and H_2_O. 

**Figure 5:**
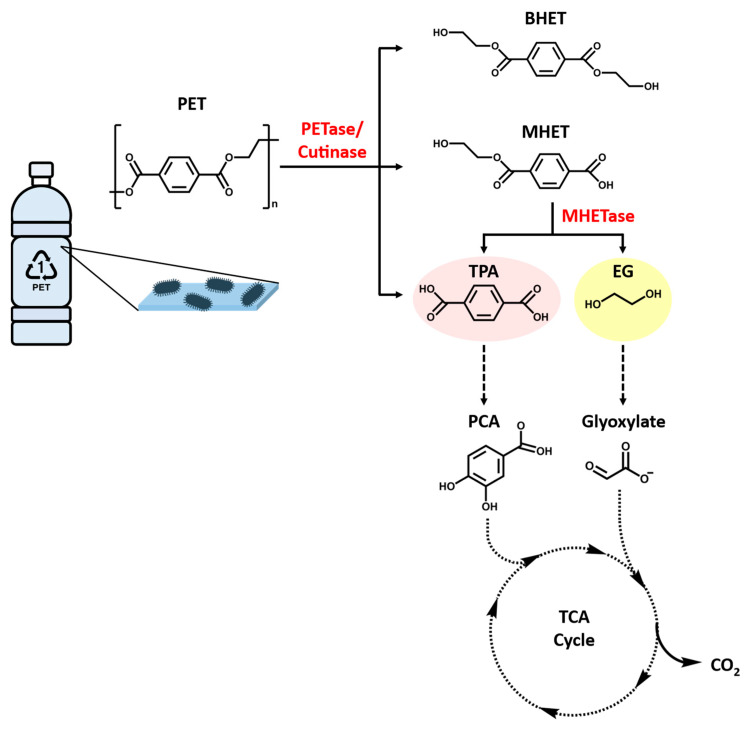
Microbial degradation of PET. PETase, PET hydrolase; MHETase, MHET hydrolase; BHET, bis(2-hydroxyethyl) terephthalic acid; MHET, mono(2-hydroxyethyl) terephthalic acid; TPA, terephthalic acid; EG, ethylene glycol; PCA, protocatechuic acid.

PET is usually regarded as non-biodegradable, but previous studies have indicated that it or its copolymers can be depolymerized by the action of hydrolytic enzymes, either in vitro [63,64] or in microbial systems [65,66]. The enzymatic hydrolysis of PET involves the release of constituent monomers terephthalic acid (TPA) and ethylene glycol (EG), which are environmentally benign monomers (Figure 5). PET can be hydrolyzed via physicochemical processes, such as glycolysis, amine decomposition, pyrolysis, and supercritical decomposition, and a biochemical process that utilizes the PET hydrolases [67]. Subsequently, the resulting PET monomers can be degraded by microorganisms endowed with the appropriate metabolic pathways as energy sources. This momentous concept to develop PET-based bioprocesses as the different pathways will generate a divergent range of metabolites, with assorted applications. For instance, TPA is converted into protocatechuic acid (PCA) that can further undergo dioxygenolytic cleavage and degradation via several different pathways prior to reach the central metabolism [68,69,70]. PCA has been used to synthesize adipic acid, an industrially important dicarboxylic acid that is a precursor to nylon 6,6, among other polymers [71,72]. Similarly, EG is assimilated via different routes based on the microorganism. For instance, in acetogen *Acetobacterium woodii*, EG is oxidized to ethanol and acetaldehyde that is eventually converted to acetate via acetyl-CoA. In other bacterial species, EG is degraded via the formation of glyoxylate [73]. It has been proposed that some strains of *Pseudomonas putida* make use of the shunt that funnels glyoxylate to the tricarboxylic acid (TCA) cycle via isocitrate or malate [74,75]. Moreover, researchers have recently discovered that a variety of microalga promote biodegradation of polymers and the energy required for degradation is reduced through the toxin system or synthesized enzymes, which are involved in a reduction of activation energy to weaken the chemical bonds in polyethylene polymers and even consume polymers as carbon source [57,58,76]. Hence, the PET degrading microorganisms and the enzymes involved are applicable for biorecycling of waste PET.

## 5. PET Recycling via Enzymatic Degradation

Compared with conventional mechanical and chemical recycling processes, biological recycling involving the enzymatic catalysis of plastic has provided many advantages nowadays. For example, the advantages of enzymatic methods, including the mild process conditions, relatively low energy input, and no need of hazardous chemicals and expensive machinery, make the enzymatic degradation a very promising option for PET recycling in future [14,16]. 

The high recalcitrant nature of plastics, including PET, is a major bottleneck for biological recycling. Enzymatic recycling of recalcitrant plastic PET has been investigated for more than two decades, and this research is now attracting more and more attention. PET monomers are linked by ester linkages, which can be hydrolyzed by various hydrolytic enzymes found in nature. Theoretically, PET is more susceptible to nature degradation than olefinic polymers, such as PE, PS, PP, and PVC [14,77,78]. The highly stable carbon–carbon bonds of the polymer backbones make these polyolefins have extremely low biodegradability, as there are no known enzymes that can directly cleave the carbon–carbon linkages. Other factors, including crystallinity of the polymer chain and hydrophobicity of the surface, also adversely affect the enzyme degradation [79]. As a semicrystalline polymer, the micro-structural arrangement of the PET consists of both amorphous and crystalline domains with a strong effect on its biodegradability. In comparison to the crystalline regions, the flexible amorphous domains of the polymer are generally more accessible to an enzymatic attack and initiate depolymerization [80]. It suggests that the biodegradation rate of plastics decreases with increasing crystallinity [81,82]. Moreover, with a high ratio of repeating aromatic terephthalate units corresponding to the limited mobility of the polymer chains, PET has extremely low biodegradability of the backbone ester linkages [83,84]. Therefore, early efforts have focused on the screening and identification of the hydrolases capable of cleaving the PET backbone ester linkages in the amorphous domain [85,86].

To date, numerous PET-hydrolyzing enzymes have been biochemically characterized, but their capacity to degrade PET and use it as a carbon source for the microbial organism has not been presented [87,88]. Enzymes displaying PET hydrolyzing activities are typically serine hydrolases and are characterized by a catalytic triad in their active site that consists of serine, histidine, and aspartate amino acids, which mainly belong to the α/β hydrolase superfamily and have evolved in a different context and for a different function [89,90]. The majority of this type of enzymes are included in the general class of carboxylic ester hydrolases (Enzyme Commission (EC) number 3.1.1), such as cutinases [91,92,93,94], lipases [95,96], and esterases [97,98], among others. These polyester hydrolases have originated from bacteria (e.g., *Thermobifida fusca*, *Thermomonospora curvata* and *Ideonella sakaiensis*) and fungi (e.g., *Fusarium solani*, *Humicola insolens* and *Aspergillus oryzae*). Most of the PET-hydrolyzing enzymes have been functionally confirmed to contain a C-terminal disulfide bond that contributes to their thermodynamic and kinetic stability [99,100,101]. From a biotechnological perspective, the discovery of PET-hydrolyzing enzymes appears to be an emerging strategy, particular with respect to the biorecycling, biocatalysis, bioremediation, and sustainable polymer modifications. 

### 5.1. PET-Hydrolyzing Enzymes from Actinobacteria

Since the discovery of a PET hydrolase, poly(butylene terephthalate-co-adipate) (BTA-1) hydrolase, from the culture supernatant of *Thermobifida fusca* in 2005 [86], various thermostable PET hydrolases and their homologs from the cutinase group (EC 3.1.1.74, a subgroup of carboxyl ester hydrolases) toward waste PET have been investigated. A variety of cutinases have been characterized from bacterial strains of thermophilic actinomycetes, especially *T. fusca*, *T. alba* [102], *T. cellulosilytica* [103], *T. curvata* [89], and *Saccharomonospora viridis* AHK190 (Cut190) [104]. Cutinases (EC 3.1.1.74) are inducible extracellular enzymes secreted by microorganisms that are capable of degrading plant cell walls [105]. Strictly speaking, cutinases are able to hydrolyze cutin, an insoluble aliphatic polyester excreted from the plant cuticle. Besides cutin, cutinases have been shown to hydrolyze various polyesters at temperature between 40–70 °C and pH 7–9, without the need for cofactors [106,107]. Notably, the substrate specificity of cutinases is broad, which exhibit hydrolytic activities for both water-insoluble long-chain triacylglycerides (typical substrates for lipases, EC 3.1.1.3) and water-soluble esters (substrates for esterases). Despite the fact that several types of lipases are also able to catalyze the PET hydrolysis, their catalytic efficiency is low [96,108,109]. In comparison with lipases, esterases usually act on esters with short-chain aliphatic region. However, only a few esterases, such as *p*-nitrobenzylesterases from *Bacillus subtilis* (BsEstB), display the hydrolytic activity on PET [110].

*Thermobifida fusca* is one of the multiple Actinobacterial strains that has been recognized as a producer of PET hydrolysis enzymes, exhibiting a remarkable degradation capability for aliphatic-aromatic copolyesters [99,111]. The possibility of enzymatic hydrolysis of commercial PET films was reported from *T. fusca* by a German research group [86]. *T. fusca* DSM 43793 has been found to produce two nearly identical extracellular hydrolases, BTA1 (commonly referred to as TfH) and BTA2, with an amino acid identity of 92% [112,113]. BTA1 has been shown to depolymerize the melt-pressed PET films from a commercial beverage bottle over three weeks at 55 °C in phosphate buffer at pH 7, with a weight loss of approximately 50% (corresponding to a weight loss of 10 mg) [86]. The degradation of the inner block of PET film was significantly higher than that of surface hydrolysable residues. These findings offered a marked improvement not only in reducing the unwanted side products but also in generating high purity of monomer for repolymerization [114]. Alignment of BTA1 and BTA2 sequences has revealed several highly conserved regions, one of which contains a Gly-His/Tyr-Ser-Met-Gly motif typical of serine hydrolase, suggesting that BTA1 and BTA2 may utilize a serine residue as a nucleophile, and an oxyanion hole formed in part by Gly and Ser residues. Moreover, BTA1 also shares a 65% similarity to a triacylglycerol lipase from *Streptomyces albus G* and 62% similarly to a triacylglycerol acylhydrolase from *Streptomyces* sp. M11 [112,115,116]. Despite the sequence homology, the function of BTA1 and lipases are significantly different. Lipases can only break ester bonds at the hydrophobic surface, however, BTA1 also exhibits an activity against dissolved esters.

The complete genomic DNA sequence of *T. fusca* YX has been determined and a number of putative esterases have been identified [117]. Two secreted triacylglycerol lipases (i.e., Tfu_0882 and Tfu_0883) display very similar enzymatic properties capable of catalyzing the breakdown of triolein and the artificial chromogenic substrate *p*-nitrophenyl butyrate (*p*NPB) with a temperature optimum around 60 °C [99,118]. A moderate thermophile, *T. fusca* KW3 (DSM 6013), has previously been shown to produce the highly hydrophobic carboxylesterases (*Tf*Cut1 and *Tf*Cut2) that are able to hydrolyze the *p*NPB, PET fibers and synthetic cyclic polyesters with an optimal activity at 60 °C and a pH of 6 [103,119,120]. However, *Tf*Cut2 exhibited almost double catalytic efficiency towards PET as compared to *Tf*Cut1 despite their high sequence identity of about 93% [99]. Another cutinase from *T. cellulolysitica* DSM44535 (Thc_Cut1 and Thc_Cut2) was cloned and characterized [95]. Thc_Cut1 and Thc_Cut2 showed distinct hydrolytic properties. Upon incubation with the 3PET, Thc_Cut1 released significantly higher amounts of soluble products TPA, MHET, hydroxyethyl benzoate, and benzoic acid than Thc_Cut2 and Thf42_Cut1 (from *T. fusca* DSM44342). When incubated with PET film, TPA was the major hydrolysis product for Thc_Cut1, whereas MHET was the most abundant product for Thc_Cut2. Furthermore, two cutin-induced *p*NPB hydrolases, Cut1 and Cut2, were originally isolated from *T. fusca* NRRL B-8184 with an optimum activity at 55 °C and pH 8.0 [118,121]. Overall, an advantage of *Thermobifida* cutinases over other cutinases is that they show higher thermostability, great tolerance of surfactant and organic solvent, versatile hydrolytic activity against a variety of synthetic polyesters, and high activity in broad pH range, which could have great biotechnological promise in many industrial applications. 

Two genes coding for the polyester hydrolases Tcur1278 and Tcur0390 by genome mining of *Thermomonospora curvata* DSM43183 were identified, which was shown to exhibit catalytic and structural features similar to enzymes from *T. fusca* and *T. cellulosilytica* [89,122]. Tcur1278 and Tcur0390 shared 62% sequence identity with BTA1. The optimal pH for both enzymes was at pH8.5. Compared to Tcur1278, Tcur0390 revealed a higher hydrolytic activity against both soluble *p*NPB and insoluble PET nanoparticles as substrates at reaction temperatures of up to 50 °C, due to the stronger substrate affinity. Tcur1278 hydrolyzed PET nanoparticles at 55 °C and 60 °C. However, both enzymes exhibited poor thermostability at their optimal reaction temperature, with an irreversible loss of more than 65% of their initial activities following incubation for 10 min.

Kawai et al. cloned a novel PET-hydrolyzing enzyme, Cut190, from thermophile *Saccharomonospora viridis* AHK190 and heterologously expressed in *Escherichia coli* Rosetta-gami B (DE3) [104]. Site-directed mutagenesis studies revealed that mutant Cut190^S226P/R228S^, with substitution of Ser226 with proline and Arg228 with serine, yielded significantly higher activity and thermostability compared with Cut190. The Cut190^S226P/R228S^ displayed the thermal activation at 50~65 °C and degraded the PET films above 60 °C. Notably, the calcium ions are required to enhance the enzyme activity and thermostability of the wild-type and mutant Cut190, which likely bind to surface acidic amino acids and not the active-site amino acids. Likewise, *Streptomyces griseus* leucine aminopeptidase is a calcium-activated and calcium-stabilized enzyme, and its activation by calcium correlates with substrate specificity, in which two surface amino acids play critical roles in modulating the enzyme via calcium [123].

Almeida et al. employed an in silico-based screening approach on the biotechnologically relevant genus *Streptomyces* to search the PETase homologs [124]. From total of 52 genomes analyzed, a candidate PETase-like gene, SM14est, was identified in *Streptomyces* sp. SM14. Alignment of SM14est and well-characterized *Is*PETase sequences indicated that the serine hydrolase motif (Gly-X1-Ser-X2-Gly) and the catalytic triad (Ser-Asp-His) were conserved in both sequences. Further molecular docking experiments showed that the SM14est possessed the capacity to bind plastics as substrates. Polyester-degrading activity of SM14est was confirmed using a polycaprolactone (PCL) plate clearing assay. Another study also found that the genome of plant pathogen *S. scabies*, the predominant causal agent of potato common scab, encodes a potential cutinase, Sub1, which was overexpressed in *E. coli* for subsequent purification and characterization [125]. Sub1was shown to be versatile because it hydrolyzes a number of natural and synthetic substrates, such as *p*-nitrophenyl esters, PET, cutin, and suberin. Additionally, the hydrolyzing activity of the Sub1 on the PET was markedly enhanced by the addition of Triton X-100 and was shown to be stable at 37 °C for at least 20 days. 

For an efficient biocatalytic PET hydrolysis, a high reaction temperature, optimally higher than the glass transition temperature (*T_g_*) of PET, is mandatory [114,126,127]. The *T_g_* value of PET is approximately 70~80 °C in air, but 10 °C lowered due to the involvement of water molecules diffusing between polymer chains; this will weaken hydrogen bonds, randomize polymer chains, and increase chain mobility. Enzymatic reactions are performed in aqueous solutions, in which *T_g_* values of PET are 60~65 °C [104]. Notably, as the temperature approaches the *T_g_* of PET, the chain mobility increases, resulting in more rapid enzymatic hydrolysis of this plastic [108]. Accordingly, most PET-hydrolyzing enzymes generally exhibit high thermostability and retain their activity at over 65 °C that might be most useful in industrial applications, which are applicable to the surface modification of PET fibers in the textile industry [96] and the breakdown process of PET polymers during chemical recycling [84]. Although thermophilic cutinases have been applied to the enzymatic recycling of PET at higher temperatures for accelerating rates of PET degradation, these cutinases could also be used for PET surface treatment at lower temperatures but tardy responses.

### 5.2. Ideonella sakaiensis Enzymes

A team of Japanese researchers have proposed three systems for PET degradation: (i) microbial consortium No. 46; (ii) *Ideonella sakaiensis* 201-F6, and (iii) a system consisting of two novel enzymes, which are applicable for the bioremediation and biorecycling of PET waste together with other potential applications, such as microplastic and microbead degradation, bioconversion, as well as PET-surface modification [128]. In order to screen PET-degrading microorganisms producing superior PET-specific degrading enzymes, it seems the more practical way is to find microorganisms that are able to grow directly on PET as carbon sources. Although various lipases, esterases, depolymerases, and cutinases have been previously reported as PET-hydrolyzing enzymes, these enzymes can only accomplish limited degradation of PET [89,97,107,109,129,130].

#### 5.2.1. Microbial Consortium No. 46

After an extensive searching of environmental samples for such PET-degrading microorganisms for over a decade, a microbial consortium No. 46 was found to both degrade PET completely and assimilate the degradation products into CO_2_ and water. Over 250 PET-debris-contaminated environmental samples, including wastewater, sediment, soil, and activated sludge, from the site surrounding a PET bottle recycling plant were screened, the microbial consortium No. 46 was isolated from one of the sediment samples collected at Sakai city, Osaka, Japan [64]. Consortium No. 46, consisting of bacteria, protozoa, and yest-like cells, was shown to adhere onto PET film during cultivation and create a drastic change in its morphology. PET film degradation occurred at a rate of 0.13 mg/cm^2^/day, with 75% of the carbon being catabolized into CO_2_ under ambient temperature conditions, which could be visualized as whitening of the PET film surface and/or decay of the PET film. Furthermore, consortium No. 46 was able to maintain its PET degradation activity for at least 10 weeks and could be re-cultivated after freezing without losing activity, which indicated the PET degradation activity of No. 46 could be retained and reproduced. 

Among the assorted kinds of bacteria, protozoa, and yeast-like cells found in the microbial consortium No. 46 as revealed by light microscopy, approximate 20 types of bacteria have been investigated. The individual roles of the identified bacteria within consortium No. 46 involved in a series of degradation process were investigated. At the beginning of the PET degradation, *Bacillus megaterium* develops a biofilm on the PET film surface. Within the biofilm, *Rhizopus* sp. cleaves the ester linkages of the PET polymer into bis(2-hydroxyethyl) terephthalate (BHET), which is further degraded into the monomers TPA and EG by *Pseudomonas* sp. Subsequently, the TPA and EG were assimilated by *Pigmentiphaga* sp. and *Mycobacterium* sp., respectively [128]. 

#### 5.2.2. *Ideonella sakaiensis* 201-F6 from Microbial Consortium No. 46

A novel Gram-negative bacterial species *Ideonella sakaiensis* 201-F6, which was later isolated from the microbial consortium No. 46, provided the basis for the next degradation system [131]. The growth of *I. sakaiensis* 201-F6 on minimal medium containing PET film has been shown to be much greater than on control medium without PET. This comparison suggested that the *I. sakaiensis* 201-F6 has an exceptionally rare ability to degrade PET as a major carbon source and energy source for its growth rather than glucose utilization.

Besides, in the liquid culture, detection of PET hydrolysis products was negligible, which indicated that *I. sakaiensis* 201-F6 is capable of completely degrading and assimilating the PET into CO_2_ as the complete oxidation product under aerobic conditions. Strain 201-F6 could degrade PET about twice as fast as the consortium No. 46 from which it was isolated. Furthermore, these unique bacterial cells were found to adhere on PET film during growth via appendages that may also assist in the delivery of secreted enzymes into the film [64,128]. Discovery of *I. sakaiensis* 201-F6 creates a potential low-energy solution for bioremediation to tackle plastic waste, which further highlights the possible value in searching for PETase-like enzymes from the *Acidovorax delafieldii* [132], *Polyangium brachysporum* [133], and *Burkholderiales* bacterium [134] as their evolutions are similar in manner. 

#### 5.2.3. Identification of PETase and MHETase in *Ideonella sakaiensis* 201-F6

The final system is based on employing the novel PET-hydrolyzing enzymes discovered in PET-assimilating bacterium, *I. sakaiensis* 201-F6. One identified open reading frame (ISF6_4831), revealed by genome sequence of *I. sakaiensis*, has been found to encode a putative lipase that shares 51% amino acid sequence identity with a TfH from *T. fusca* [86,128]. The corresponding recombinant *I. sakaiensis* proteins created cater-like pitting on the PET film surface and released the PET degradation products into aqueous medium. This *I. sakaiensis* cutinase-like enzyme, namely PETase or *Is*PETase, was subsequently assigned to the new EC 3.1.1.101. The mono(2-hydroxyethyl) terephthalic acid (MHET) is the major product released by the *Is*PETase, together with minor amounts of TPA and BHET. In comparison with the hydrolytic activities toward *p*-nitrophenol-linked aliphatic esters and PET among the known PET-hydrolyzing enzymes, such as TfH, leaf-branch compost cutinase (LCC) [92] and *F. solani* fungal cutinase (FsC) [135], *Is*PETase has been shown to have the highest catalytic preference for PET but the lowest hydrolytic activity for aliphatic esters. After 18 h incubation at 30 °C and pH7.0, the activity of *Is*PETase against low-crystallinity (1.9%) PET film was assessed to be 120, 5.5, and 88 times as high as that of TfH, LCC, and FsC, respectively [64]. Likewise, the *Is*PETase was also more active than TfH, LCC, and FsC against highly crystallized, commercial bottle-derived PET (hcPET) in pH9.0 bicine-NaOH buffer for 18 h at 30 °C, even though the hcPET greatly reduces the enzymatic hydrolysis of its ester linkages. The *Is*PETase displaying the highest substrate specificity and prominent hydrolytic activity for PET at ambient temperature makes it a promising candidate for new biodegradation strategies. However, *Is*PETase accomplishes the PET degradation well under moderate (mesophilic) temperature conditions (between 20~40 °C) but is heat-labile, whereas the other PET-hydrolyzing enzymes are optimally active at higher temperatures owing to their thermophilicity. 

**Figure 6:**
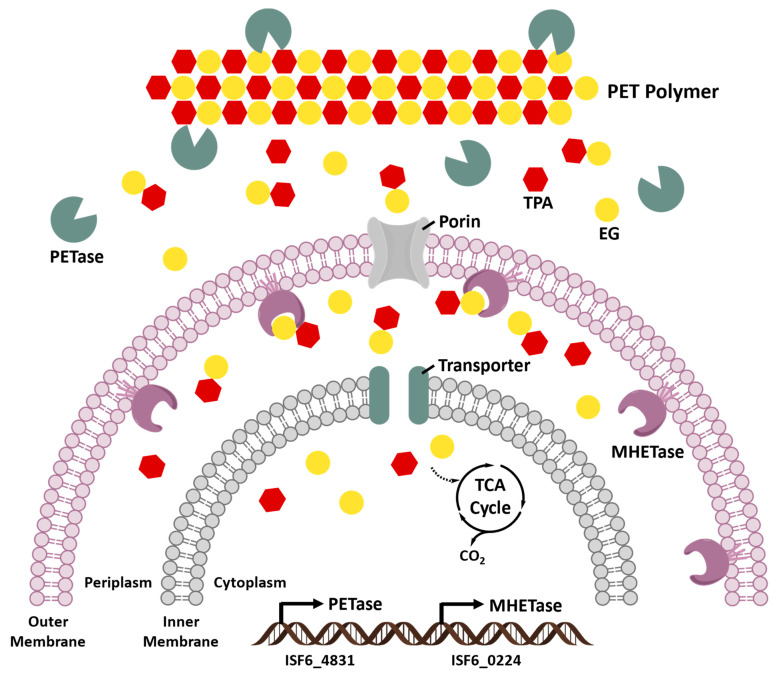
PET metabolic pathway by *Ideonella sakaiensis* [128].

The primary product of *Is*PETase hydrolysis is MHET, which is broken down into the monomers, TPA and EG, by a second enzyme (ISF6_0224) identified from *I. sakaiensis*. This enzyme was designated as MHET hydrolase (termed MHETase) and was also assigned a new EC number (3.1.1.102) [64]. MHETase is a member of the tannase family and has been shown to efficiently hydrolyze MHET with a turnover rate (*k_cat_*) of 31 ± 0.8 S^−1^ and Michaelis constant (*K_m_*) of 7.3 ± 0.6 μM, but it displays little activity against PET, BHET, aliphatic esters, or typical aromatic ester compounds. The biochemical properties of PETase and MHETase, along with their predicted localization, revealed the PET metabolic mechanism by *I. sakaiensis* (Figure 6). *Is*PETase and MHETase catalyze similar reactions in different locations. *Is*PETase, which acts extracellularly, is responsible for hydrolytic conversion of PET into oligomers that include MHET as the major component and TPA. The PET hydrolysates are then transported into the periplasmic space via an outer membrane protein (e.g., porin) and MHETase further hydrolyzes MHET into PET monomers, TPA and EG. MHETase is predicted to be an outer membrane anchored lipoprotein [64]. Interestingly, a gene cluster in *I. sakaiensis* is highly identical with two TPA degradation gene clusters identified in *Comamonas* sp. strain E6 [136]. The expression of this cluster in *I. sakaiensis* is significantly upregulated under the presence of TPA. Subsequently, TPA is taken up into the cytoplasm via TPA transporter coupled with a TPA-binding protein [137], and then enters the central TCA cycle via protocatechuic acid (PCA) [138,139]. Likewise, EG is also metabolized in the TCA cycle via glyoxylate [74,140]. 

Integration of protein sequence and structure information is essential in providing a good starting point for protein engineering, which may contribute to the enzyme redesigning to make them more amenable for industrial applications. The enzyme modifications can facilitate the discovery of new paths of molecular evolution, designing of efficient enzymes, locating active sites and crucial residues, shifting the substrate specificity, and changing cofactor specificity [141]. Crystal structures of recombinant PETase have been determined in several groups [88,142,143]. Three-dimensional structures of *Is*PETase reveal the features shared by bacterial lipases and cutinases, along with unique characteristics that differentiate the enzyme from cutinases. *Is*PETase shares ~50% amino acid identity with those of cutinases from *T. fusca* KW3 (*Tf*Cut2), *S. viridis* (Cut190), and *T. alba* (*Ta*Cut) [144]. As predicted from the sequence homology to the lipase and cutinase families, *Is*PETase adopts the canonical α/β-hydrolase fold and employs the catalytic triad residues consisting of Ser160, His237, and Asp206, suggesting a charge-relay system [88,128,145]. Notably, *Is*PETase has a highly polarized surface charge, creating a dipole across the molecule (isoelectric point (pI) of 9.6); whereas *T. fusca* cutinase, in common with other cutinases, has a number of small patches of both acidic and basic residues distributed over the surface (pI of 6.3) [88]. Furthermore, in light of recent studies that suggest MHETase, the second key enzyme isolated from *I. sakaiensis*, possesses a α/β-hydrolase domain essential for catalysis and a lid domain conferring substrate specificity, and is predicted to be a fairly acidic protein (pI of 5.2) [146].

In terms of the active site, relative broadening of the active-site cleft is observed in *Is*PETase (cleft width was calculated from the distance between the van der Waals surface of Thr88 and Ser238, 8.46 Å) in comparison with the *T. fusca* cutinase (distance between the Thr61 and Phe209, 2.98 Å), suggesting *Is*PETase provides more space to accommodate PET as a substrate and enabling more efficient PET depolymerization [88]. Another striking difference between *Is*PETase and the closest cutinase homologs is the existence of two disulfide bonds in *Is*PETase, one adjacent to the active-site and one near the C terminus of the protein. Almost all of the known bacterial cutinases have one disulfide bond near the C terminus; the disulfide bond is formed between Cys281 and Cys299 in *Tf*Cut2, between Cys287 and Cys302 in Cut190, and between Cys276 and Cys294 in *Ta*Cut. This disulfide bond is also conserved in *Is*PETase formed between Cys273 and Cys289 near the C terminus, and an additional one is between Cys203 and Cys239 at the vicinity of the active site [147]. In order to understand the effect of the additional disulfide bond near the active site, Cys203 and Cys239 were substituted by alanine (*Is*PETase^C203A/C239A^). Taniguchi et al. (2009) demonstrated that the activity and melting temperature (*T_m_*) were markedly decreased in the double mutation. This finding supported the importance of the presence of active-site disulfide for the thermal stability of *Is*PETase.

Interestingly, other significant structural differences were also observed between *Tf*Cut2 and *Is*PETase, namely that His169 and Phe249 residues in *Tf*Cut2 are located at the corresponding positions of Trp159 and Ser238 in *Is*PETase. To determine the role of these two residues, Trp159 and Ser238 residues were replaced with His and Phe, respectively. The *Is*PETase^W159H^ and the *Is*PETase^S238F^ variants exhibited dramatically decreased hydrolytic activities from both uses of BHET and PET as substrate [144]. Through site-directed mutagenesis, Trp159 and Ser238 residues play a crucial role in the high PET-degrading activity of *Is*PETase. Surprisingly, in contrast to the single mutant, narrowing the binding cleft via mutation of these two active-site residues to conserved amino acids (*Is*PETase^W159H/S238F^) in cutinase-like active-site cleft accommodates more productive substrate-binding interaction and enables improved PET and poly(ethylene furanoate) (PEF) degradation capacity [88]. Overall, these findings open up an exciting platform for protein engineering of PETase to increase the efficiency and substrate range, as well as to provide clues of how to further engineer thermophilic cutinases in practical industrial applications.

### 5.3. PET-Hydrolyzing Enzymes from Fungi

Although bacterial PET-hydrolyzing enzyme catalytic properties and mechanism are similar to the fungal cutinase, sequential and structural differences place the *T. fusca* enzymes into a different subfamily of cutinase from the fungal enzymes. Cutinases from some phytopathogenic and non-phytopathogenic fungi, such as *Fusarium solani pisi* [148,149,150], *Humicola insolens* [107], *Botrytis cinerea* [151], *Colletotrichum gloeosporioides* [152], *Monilinia fructicola* [153,154], *Magnaporthe grisea* [155], *Aspergillus oryzae* [156], and *Thielavia terrestris* [157,158], have been investigated by biochemical and structural approaches. The fungal cutinases have been shown to be critical for pathogen–host compatible interaction due to the disruption of some cutinase genes’ decreasing virulence or eliminating pathogenicity on host plants [159,160,161,162]. Most fungi have multiple copies of cutinase genes and the role of each cutinase gene in disease could be different. For instance, Sweigard et al. have identified a cutin-degrading enzyme, encoded by the *M. grisea CUT1*, that was previously shown to be dispensable for pathogenicity [163]. However, the expression of *M. grisea CUT2* was dramatically upregulated during infection. Disruption of *CUT2* displayed anomalous germling morphology and severely impaired host penetration, thus drastically attenuated fungal virulence [164]. Morphological and pathogenicity defects in the *CUT2* mutant could be fully restored to wild-type levels by adding pharmacological activators of the cAMP-protein kinase A and diacylglycerol-protein kinase C signaling cascades [164,165].

*Fusarium solani pisi* cutinase (FsC) was the first to be biochemically characterized in detail, especially after its cloning and expression in a heterologous host, which also led to the determination of its three-dimensional structure [166,167]. The catalytic activities of three different cutinases from filamentous fungus *H. insolens* (HiC), *F. solani pisi* (FsC), and bacterium *Pseudomonas mendocina* (PmC) have been previously studied on low-crystallinity and biaxially oriented PET films as model substrates, containing 7% and 35% crystallinity, respectively [107]. Cutinases exhibited about 10-fold higher activity for the low-crystallinity than for the biaxially oriented PET films, which were assayed using a pH-stat to measure sodium hydroxide consumption that directly monitors released acid during ester cleavage [107]. This result is consistent with previous reports by many others using different enzyme–polymer systems, an increase in PET crystallinity negatively affects the PET degradation rate due to decreased polymer chain mobility [80,168,169,170]. Moreover, incubation of HiC with low-crystallinity PET films resulted in 97% film weight loss at 70 °C within 96 h [107]. In contrast, only 5% weight loss was achieved by the FsC and PmC on the low-crystallinity PET films at 40 °C and at 50 °C, respectively. The reason for HiC’s significant high activity for PET hydrolysis is largely attributed to the higher thermal stability of HiC, which is close to the *T_g_* value of the PET. By remaining active at the PET’s *T_g_*, HiC benefits from higher mobility of the chains in the amorphous phase, resulting in increasing the accessibility of HiC to PET ester groups. Therefore, the potential to completely convert commercial low crystallinity PET materials to water-soluble products was demonstrated. Indeed, most of the PET recycling is concentrated in the bottle manufacturing industry, which uses low crystallinity PET to achieve high bottle transparency [86,171].

Generally, the stability of free cutinase is poor, thus the soluble enzymes have to be immobilized to be reused for long lengths of time in industrial reactors [172]. Several polymers are used as carrier materials in the enzyme immobilization to achieve the highest possible catalytic activity of a biocatalyst [173]. For instance, FsC was immobilized on a biomimetic triazine-scaffolded synthetic affinity ligand to achieve enzyme stabilization. This ligand was able to strongly bind FsC and led to an impressive 57-fold increase in half-life as compared with the free FsC at 60 °C for 34 h [174]. Similarly, the immobilization of FsC onto magnetic chitosan beads, using genipin as the crosslinker, exhibited outstanding recyclability as it could maintain more than 50% residual activity after 10 cycles [175]. Another *Fusarium oxysporum* cutinase loaded nanoporous gold-polyethyleneimine has been fabricated for the application in simultaneous removal of di(2-ethylhexyl)phthalate and Pb(II) from contaminated water, which can be used more than five times before regeneration [176]. Furthermore, the extracellular cutinase of *Aspergillus* sp. RL2Ct was stabilized in an active conformation by immobilization on acrylamide-grafted copolymer of chitosan and chitin [172]. This immobilized cutinase exhibited enhanced thermal stability and improved efficiency, which can be recycled up to 64 times without considerable loss of activity. The improved thermal stability of the immobilized cutinase indicates that the polymer as matrix provides conformational stability to the enzyme upon immobilization as the free enzyme gains thermal inactivation easily, owing to breaking of intermolecular forces responsible for the three-dimensional structure required for its catalytic activity [177,178].

Besides cutinase, lipase has also been used by several researchers for PET hydrolysis. Effective degradation of polyester-nanoparticles using lipases from yeast *Candida cylindracea* (CcL) and bacterium *Pseudomonas* sp. (PsL) has been previously reported [179]. By employing the BHET inducer to the culture medium, the extracellular lipase activity produced by *A. oryzae* was directed toward the hydrolysis of PET [180]. Moreover, using PET and BHET as substrates revealed *Candida antarctica* lipase B (CALB) and *H. insolens* cutinase (HiC) as potential biocatalysts [181]. CALB showed completely converted BHET to TPA, although lower efficiency toward initial PET biodegradation. In contrast, HiC demonstrated better performance for PET hydrolysis, but accumulated considerable amounts of the MHET intermediate. However, the combination of CALB and HiC enzymes synergistically improve the complete PET depolymerization to TPA, resulting in a 7.7-fold increase in TPA yield from PET [181,182]. 

### 5.4. Metagenome-Derived PET-Hydrolyzing Enzymes

Metagenomics offer an enormous opportunity to understand and discover the functions that the uncultured fraction of microbes bring to the environment [183]. Advances in metagenomic analysis have created the possibility of obtaining complete or nearly complete genome sequences from uncultured microorganisms, broadening the extent to which genetic information can be explored [184,185]. Currently, only a small number of microbial genera and enzymes have been described to possess the ability to degrade PET. Danso et al. developed a search algorithm that identified over 800 putative PET hydrolase candidate genes through screening the existing genome and metagenome databases across the marine and terrestrial [186]. From the obtained homologous sequences, 13 potential PET hydrolase homologs, namely PET1–PET13, were chosen due to their sequence similarities to known PET hydrolase. Four novel PET hydrolase genes (PET2, PET5, PET6, and PET12) were functionally verified by cloning and expressing them heterologously in *E. coli*, with active clones producing halos on PET nanoparticles or PCL. Among these active enzymes, PET2 was derived from a marine metagenomics dataset [187], and PET6 was derived from *Vibrio gazogenes* strain DSM-21264 [188]. Both enzymes showed traits of thermostability, with optimal temperature at 70 °C for PET2 and 55 °C for PET6. Remarkably, PET2 retained 80% of its relative activity at 90 °C after incubation for over 5 h. Furthermore, all of the newly identified PET hydrolases originated mainly from three bacterial phyla, *Proteobacteria*, *Actinobacteria*, and *Bacteroidetes*. It is worth noting that the Bacteroides have not been associated with PET depolymerization to date, but the Bacteroides have been increasingly regarded as very efficient degraders of other polymers [189,190,191].

Sulaiman et al. identified that the gene encoding a novel cutinase homolog, leaf-branch compost cutinase (LCC), was cloned from the leaf-branch compost metagenome library by functional screening using tributyrin agar plates [92]. LCC showed the amino acid sequence identity of 59.7% to *T. curvata* lipase and 57.4% to *T. fusca* cutinase [145]. LCC was heterologously overexpressed in *E. coli* and exhibited the activity to hydrolyze various fatty acid monoesters with acyl chain lengths 2 to 18, with a preference for short-chain substrates (C_4_) most optimally at pH 8.5 and 50 °C [92]. LCC also had an ability to degrade the PET and PCL films, at a rate of 12 mg/h/mg and 300 mg/h/mg of enzyme, respectively. Moreover, when using crystalline bottle-grade PET as a substrate and the temperature of system was 65 °C, the PET degradation rate of LCC (93.2 mg/h/mg of enzyme) has outperformed most other enzymes, such as *T. fusca* BTA1 and BTA2, *F. solani pisi* FsC, and *I. sakaiensis Is*PETase, by at least 33-fold [94]. However, the decomposition rate of PET by LCC remains insufficient to catch up with the production rate of plastic waste. 

Owing to the high potential of the LCC enzyme in degrading persistent semi-aromatic polyesters such as PET, it inspired more efforts to seek some strategies to make the LCC work more efficiently. Detailed structural and functional analysis of LCC facilitates understanding of the mechanism by which LCC hydrolyzes PET and thereby leads to the development of a more efficient enzyme. In order to improve both the activity and the thermostability of LCC, Tournier et al. [94] generated all 209 possible variants through site-specific saturation mutagenesis. Of such LCC mutant variants, two variants, namely ICCG (F243I/D238C/S283C/Y127G) and WCCG (F243W/D238C/S283C/Y127G), demonstrated improved deactivation temperatures and increased PET depolymerization by 82% and 85% in 20 h and 15 h at pH 8 and 72 °C, respectively. Compared with ICCG and WCCG variants, wild-type LCC showed the reduced thermostability and only 53% PET depolymerization at 20 h. Moreover, with an enzyme concentration of 3 milligrams per gram of PET, both ICCG and WCCG variants achieved a minimum of 90% PET depolymerization and a mean TPA productivity of 16.7 g/L/h over 10 h. By further optimizing the developed process, 863 kg of TPA were recycled from 1 ton of PET waste, which were further successfully re-used to synthesize the virgin PET after purification. Bottles blown from this kind of PET had similar mechanical properties to those of commercial bottles. This progress is an excellent example addressing the issue of PET plastic disposal to achieve sustainable goals and the idea of a circular economy.

In view of the high specificity of the enzyme, although the LCC variants have exhibited superior biodegradability, it is not applicable to degrade other types of non-ester polymers (e.g., PE, PP and PS) [192]. From this perspective, it is necessary to discovery other enzymes for different types of plastics through metagenomic tools and optimize their properties through enzyme engineering.

## 6. Future Opportunities and Challenges for Biorecycling of PET Plastic

Biorecycling can be achieved via both microbial degradation and enzymatic degradation processes, followed by a further chemical or biological conversion of the degraded monomers into polymers or other value-added chemicals. Biodegradation is limited by the organisms and the enzymes used, inherent polymer properties, and the choice of pre-treatment of plastics. The future success of this process will rely on optimization and/or modification of these factors. 

The advances in in modern biology and biotechnology have brought great opportunities for creating transformative biorecycling strategies in future. First, new synthetic biology tools and metabolic engineering strategies provide a great potential to reengineer and improve the currently identified microorganisms that can efficiently decompose the solid plastic wastes and directly use the degraded products as the carbon source for biomanufacturing. Second, site-directed mutagenesis has been routinely used to redesign enzymes, but the achievements of this technology basically depend on the availability of three-dimensional protein structures. Numerous PET-hydrolyzing enzymes have been successfully enhanced with enzyme engineering technologies [88]. Third, new protein engineering strategies, such as direct evolution and AI-guided protein design and mutation, may provide more opportunities to generate novel enzymes that have extremely high activity, specificity, stability, thermal tolerance, and resistance to inhibitors or impurities [141]. Other techniques based on cross-referenced plastic substrate structures and comparison with protein alignment data have also been used. 

However, several challenges still remain to achieve the biorecycling of PET and other types of plastic wastes at large scale. First, the impact of diffident physical properties of PET on biodegradation efficiency is profound and needs to be further studied to design a more efficient biodegradation process. PET plastics are inherently difficult to bond and coat because they are hydrophobic, non-polar materials, chemically inert, and have poor surface wettability. Since biodegradation of polymers is a surface process, the adsorption of enzymes on the surface of plastics may play an important role. To overcome the limitations in the process, we can either engineer the enzyme to make it more suitable for a specific type of plastic or pretreat the plastic materials to improve the properties for biodegradation. For example, *Is*PETase is highly specific to PET, however, it shows poor catalytic activity and is difficult to be used in industrial processes. Besides enzyme engineering, pre-treatment of PET plastics provides a feasibility to promote the interaction between the enzymes and plastics. It was found that the hydrolytic activity of PETase could be improved by 120-fold by surface coating of the low-crystallinity PET film with anionic surfactants [193]. The binding of surfactants to the PET film makes the surface anionic, thereby recruiting more cationic PETase. 

The second major challenge is the uncertainties in process conditions and the requirements for the pretreatments that are caused by the variations in PET waste sources. The PET wastes from different applications may have significantly different impurities and physical properties, such as shape, crystallinity, glass transition temperature, and mechanic strength, which may lead to significantly different biodegradation efficiency under the same process and reaction conditions. Further study on different sources of PET should be conducted. 

The third major challenge is the lack of enough knowledge and experience in scaling up current biodegradation technology. There is still no or little research on the reaction engineering and reactor design studies for major factors or parameters that are critical for the scale-up process. For example, large quantities of TPA released from the PET degradation may be insoluble in the reaction solution, we expect that avoiding the limitations in mixing and mass transfer in the enzymatic degradation of PET should be addressed so that we can eventually develop a biorecycling process with efficient degradation, product removal, and product purification at commercial scale. In addition, production of enzymes at high productivity should also be investigated to minimize the enzyme cost. 

## 7. Conclusions

PET is the most abundant polyester manufactured globally and it is typically recycled at a rate of nearly 25%. Although this is the highest rate of all plastic categories, product quality and supply chain economics are still limitations. Many end applications of PET are not suited to closed-loop recycling, thus a significant portion of used PET leaks into the environment. The need for improved PET plastic circularity is obvious, and much work has been devoted to dealing with this challenge for several decades. Currently, mechanical and chemical recycling are the two major recycling methods for PET, but the former may lead to lower quality as compared to the original plastic and the latter faces new challenges in additional economic and environmental costs. Fortunately, recent advances in modern biotechnology opens a door for potentially a more sustainable and economical solutions. Biorecycling of PET can be achieved by biodegradation with either engineered microorganisms or enzymes under mild reaction conditions, followed by further recycling of the degraded monomers to PET or upcycling them to other more valuable products. Enzymatic degradation of PET by using an engineered enzyme, such as leaf-branch compost cutinase (LCC), may achieve 90% or higher degradation in two days or shorter time for certain amorphas PET. The new possibilities for the bioconversion of post-consumer PET plastics into environmentally benign monomers, as discussed in this review, can be used as drop ins at existing industrial facilities. Though significant progresses have been made to overcome the technological hurdles for biodegradation, several challenges still remain to commercialize biorecycling technology, including process scale up and further improvements in both biocatalyst activity and process robustness. It is our expectation that the prospects for a circular bio-based economy of PET will eventually lead to a pathway to a sustainable plastic industry in the future. 

## Data Availability

The data presented in this study are available on request from the corresponding author.

## References

[1] Saminathan P., Sripriya A., Nalini K., Sivakumar T., Thangapandian V. (2014). Biodegradation of plastics by *Pseudomonas putida* isolated from garden soil samples. J. Adv. Bot. Zool..

[2] Ahmed T., Shahid M., Azeem F., Rasul I., Shah A.A., Noman M., Hameed A., Manzoor N., Manzoor I., Muhammad S. (2018). Biodegradation of plastics: Current scenario and future prospects for environmental safety. Environ. Sci. Pollut. Res..

[3] Andrady A.L. (2015). Plastics and Environmental Sustainability.

[4] Nkwachukwu O.I., Chima C.H., Ikenna A.O., Albert L. (2013). Focus on potential environmental issues on plastic world towards a sustainable plastic recycling in developing countries. Int. J. Ind. Chem..

[5] Geyer R., Jambeck J.R., Law K.L. (2017). Production, use, and fate of all plastics ever made. Sci. Adv..

[6] Hopewell J., Dvorak R., Kosior E. (2009). Plastics recycling: Challenges and opportunities. Philos. Trans. R. Soc. B Biol. Sci..

[7] Zhao S., Wang T., Zhu L., Xu P., Wang X., Gao L., Li D. (2019). Analysis of suspended microplastics in the Changjiang Estuary: Implications for riverine plastic load to the ocean. Water Res..

[8] Hundertmark T., Mayer M., McNally C., Simons T.J., Witte C. How Plastics Waste Recycling Could Transform the Chemical Industry. https://search-ebscohost-com.umasslowell.idm.oclc.org/login.aspx?direct=true&db=ply&AN=20190602564&site=eds-live.

[9] Rochman C.M., Browne M.A., Halpern B.S., Hentschel B.T., Hoh E., Karapanagioti H.K., Rios-Mendoza L.M., Takada H., Teh S., Thompson R.C. (2013). Classify plastic waste as hazardous. Nature.

[10] Rochman C.M., Hoh E., Kurobe T., Teh S.J. (2013). Ingested plastic transfers hazardous chemicals to fish and induces hepatic stress. Sci. Rep..

[11] Wilcox C., Van Sebille E., Hardesty B.D. (2015). Threat of plastic pollution to seabirds is global, pervasive, and increasing. Proc. Natl. Acad. Sci. USA.

[12] Sussarellu R., Suquet M., Thomas Y., Lambert C., Fabioux C., Pernet M.E.J., Le Goïc N., Quillien V., Mingant C., Epelboin Y. (2016). Oyster reproduction is affected by exposure to polystyrene microplastics. Proc. Natl. Acad. Sci. USA.

[13] Narancic T., O’Connor K.E. (2019). Plastic waste as a global challenge: Are biodegradable plastics the answer to the plastic waste problem?. Microbiology.

[14] Webb H.K., Arnott J., Crawford R.J., Ivanova E.P. (2013). Plastic degradation and its environmental implications with special reference to poly (ethylene terephthalate). Polymers.

[15] Kint D., Muñoz-Guerra S. (1999). A review on the potential biodegradability of poly (ethylene terephthalate). Polym. Int..

[16] Awaja F., Pavel D. (2005). Recycling of PET. Eur. Polym. J..

[17] Karayannidis G.P., Psalida E.A. (2000). Chain extension of recycled poly (ethylene terephthalate) with 2,2′-(1,4-phenylene)bis(2-oxazoline). J. Appl. Polym. Sci..

[18] Scheirs J. (1998). Polymer Recycling: Science, Technology and Applications.

[19] Caldicott R.J. (1999). The basics of stretch blow molding PET containers. Plast. Eng..

[20] Rosato D.V. (2012). Plastics Processing Data Handbook.

[21] Hiraga K., Taniguchi I., Yoshida S., Kimura Y., Oda K. (2019). Biodegradation of waste PET: A sustainable solution for dealing with plastic pollution. EMBO Rep..

[22] Barnes D.K., Galgani F., Thompson R.C., Barlaz M. (2009). Accumulation and fragmentation of plastic debris in global environments. Philos. Trans. R. Soc. B Biol. Sci..

[23] Gregory M.R. (2009). Environmental implications of plastic debris in marine settings—Entanglement, ingestion, smothering, hangers-on, hitch-hiking and alien invasions. Philos. Trans. R. Soc. B Biol. Sci..

[24] Oehlmann J., Schulte-Oehlmann U., Kloas W., Jagnytsch O., Lutz I., Kusk K.O., Wollenberger L., Santos E.M., Paull G.C., Van Look K.J. (2009). A critical analysis of the biological impacts of plasticizers on wildlife. Philos. Trans. R. Soc. B Biol. Sci..

[25] Jambeck J.R., Geyer R., Wilcox C., Siegler T.R., Perryman M., Andrady A., Narayan R., Law K.L. (2015). Plastic waste inputs from land into the ocean. Science.

[26] McArthur E. (2017). The New Plastics Economy: Rethinking the Future of Plastics & Catalysing Action.

[27] Zhang H., Wen Z.-G. (2014). The consumption and recycling collection system of PET bottles: A case study of Beijing, China. Waste Manag..

[28] Prata J.C., Silva A.L.P., Da Costa J.P., Mouneyrac C., Walker T.R., Duarte A.C., Rocha-Santos T. (2019). Solutions and integrated strategies for the control and mitigation of plastic and microplastic pollution. Int. J. Environ. Res. Public Health.

[29] Schneider D.R., Ragossnig A. (2015). Recycling and incineration, contradiction or coexistence?. Waste Manag. Res..

[30] Calcott P., Walls M. (2000). Can downstream waste disposal policies encourage upstream “design for environment”?. Am. Econ. Rev..

[31] Teuten E.L., Saquing J.M., Knappe D.R., Barlaz M.A., Jonsson S., Björn A., Rowland S.J., Thompson R.C., Galloway T.S., Yamashita R. (2009). Transport and release of chemicals from plastics to the environment and to wildlife. Philos. Trans. R. Soc. B Biol. Sci..

[32] Liu Z., Adams M., Walker T.R. (2018). Are exports of recyclables from developed to developing countries waste pollution transfer or part of the global circular economy?. Resour. Conserv. Recycl..

[33] Arena U. (2012). Process and technological aspects of municipal solid waste gasification. A review. Waste Manag..

[34] Lombardi L., Carnevale E., Corti A. (2015). A review of technologies and performances of thermal treatment systems for energy recovery from waste. Waste Manag..

[35] Zevenhoven R., Karlsson M., Hupa M., Frankenhaeuser M. (1997). Combustion and gasification properties of plastics particles. J. Air Waste Manag. Assoc..

[36] Gilpin R.K., Wagel D.J., Solch J.G. (2003). Production, Distribution, and Fate of Polychlorinated Dibenzo-p-dioxins, Dibenzofurans and Related Organohalogens in the Environment. Dioxins and Health.

[37] Curlee T.R., Das S. (1991). Plastic Wastes: Management, Control, Recycling and Disposal.

[38] Okan M., Aydin H.M., Barsbay M. (2019). Current approaches to waste polymer utilization and minimization: A review. J. Chem. Technol. Biotechnol..

[39] Royte E. Is Burning Plastic Waste a Good Idea?. https://www.nationalgeographic.com/environment/article/should-we-burn-plastic-waste.

[40] Achilias D.S. Chemical recycling of polymers. The case of poly (methyl methacrylate). Proceedings of the International Conference on Energy & Environmental Systems.

[41] Saphire D., Bluestone M. (1994). Case Reopened: Reassessing Refillable Bottles.

[42] Feron V., Jetten J., De Kruijf N., Van Den Berg F. (1994). Polyethylene terephthalate bottles (PRBs): A health and safety assessment. Food Addit. Contam..

[43] Schmidt L. (1998). PET recycling: The view from NAPCOR. Resour. Recycl..

[44] Albrecht P., Brodersen J., Horst D., Scherf M. (2011). Reuse and Recycling Systems for Selected Beverage Packaging from a Sustainability Perspective.

[45] Paszun D., Spychaj T. (1997). Chemical recycling of poly (ethylene terephthalate). Ind. Eng. Chem. Res..

[46] Burillo G., Clough R.L., Czvikovszky T., Guven O., Le Moel A., Liu W., Singh A., Yang J., Zaharescu T. (2002). Polymer recycling: Potential application of radiation technology. Radiat. Phys. Chem..

[47] Majauskienė R., Miknius L. (2015). Properties of residual marine fuel produced by thermolysis from polypropylene waste. Mater. Sci..

[48] Dimitris S., Achilias L. (2014). Recent advances in the chemical recycling of polymers (PP, PS, LDPE, HDPE, PVC, PC, Nylon, PMMA). Mater. Recycl. Trends Perspect.

[49] Jagtap M., Khatavkar M., Quazi T. (2015). Methods for waste plastic recycling. Int. J. Recent Technol. Mech. Electr. Eng..

[50] Sinha V., Patel M.R., Patel J.V. (2010). PET waste management by chemical recycling: A review. J. Polym. Environ..

[51] Garcia J.M., Robertson M.L. (2017). The future of plastics recycling. Science.

[52] Al-Salem S.M., Lettieri P., Baeyens J. (2009). Recycling and recovery routes of plastic solid waste (PSW): A review. Waste Management.

[53] Schulte P.A., McKernan L.T., Heidel D.S., Okun A.H., Dotson G.S., Lentz T.J., Geraci C.L., Heckel P.E., Branche C.M. (2013). Occupational safety and health, green chemistry, and sustainability: A review of areas of convergence. Environ. Health.

[54] Idumah C.I., Nwuzor I.C. (2019). Novel trends in plastic waste management. SN Appl. Sci..

[55] Mourshed M., Masud M.H., Rashid F., Joardder M.U.H. (2017). Towards the effective plastic waste management in Bangladesh: A review. Environ. Sci. Pollut. Res..

[56] Glaser J.A. (2019). Biological degradation of polymers in the environment. Plastics in the Environment.

[57] Khoo K.S.H., Ho L.Y., Lim H.R., Leong H.Y., Chew K.W. (2021). Plastic waste associated with the COVID-19 pandemic: Crisis or opportunity?. J. Hazard. Mater..

[58] Chia W.Y., Ying Tang D.Y., Khoo K.S., Kay Lup A.N., Chew K.W. (2020). Nature’s fight against plastic pollution: Algae for plastic biodegradation and bioplastics production. Environ. Sci. Ecotechnol..

[59] Lucas N., Bienaime C., Belloy C., Queneudec M., Silvestre F., Nava-Saucedo J.-E. (2008). Polymer biodegradation: Mechanisms and estimation techniques—A review. Chemosphere.

[60] Dussud C., Ghiglione J.-F. (2014). Bacterial degradation of synthetic plastics. CIESM Workshop Monographs.

[61] Jaiswal S., Sharma B., Shukla P. (2020). Integrated approaches in microbial degradation of plastics. Environ. Technol. Innov..

[62] Shah A.A., Hasan F., Hameed A., Ahmed S. (2008). Biological degradation of plastics: A comprehensive review. Biotechnol. Adv..

[63] Barth M., Wei R., Oeser T., Then J., Schmidt J., Wohlgemuth F., Zimmermann W. (2015). Enzymatic hydrolysis of polyethylene terephthalate films in an ultrafiltration membrane reactor. J. Membr. Sci..

[64] Yoshida S., Hiraga K., Takehana T., Taniguchi I., Yamaji H., Maeda Y., Toyohara K., Miyamoto K., Kimura Y., Oda K. (2016). A bacterium that degrades and assimilates poly (ethylene terephthalate). Science.

[65] Nowak B., Pająk J., Łabużek S., Rymarz G., Talik E. (2011). Biodegradation of poly (ethylene terephthalate) modified with polyester “Bionolle^®^” by Penicillium funiculosum. Polimery.

[66] Sharon C., Sharon M. (2012). Studies on biodegradation of polyethylene terephthalate: A synthetic polymer. J. Microbiol. Biotechnol. Res..

[67] Kawai F., Kawabata T., Oda M. (2020). Current state and perspectives related to the polyethylene terephthalate hydrolases available for biorecycling. ACS Sustain. Chem. Eng..

[68] Frazee R.W., Livingston D., LaPorte D., Lipscomb J. (1993). Cloning, sequencing, and expression of the Pseudomonas putida protocatechuate 3, 4-dioxygenase genes. J. Bacteriol..

[69] Kasai D., Fujinami T., Abe T., Mase K., Katayama Y., Fukuda M., Masai E. (2009). Uncovering the protocatechuate 2, 3-cleavage pathway genes. J. Bacteriol..

[70] Maruyama K., Shibayama T., Ichikawa A., Sakou Y., Yamada S., Sugisaki H. (2004). Cloning and characterization of the genes encoding enzymes for the protocatechuate meta-degradation pathway of Pseudomonas ochraceae NGJ1. Biosci. Biotechnol. Biochem..

[71] Johnson C.W., Salvachúa D., Khanna P., Smith H., Peterson D.J., Beckham G.T. (2016). Enhancing muconic acid production from glucose and lignin-derived aromatic compounds via increased protocatechuate decarboxylase activity. Metab. Eng. Commun..

[72] Salvador M., Abdulmutalib U., Gonzalez J., Kim J., Smith A.A., Faulon J.-L., Wei R., Zimmermann W., Jimenez J.I. (2019). Microbial genes for a circular and sustainable bio-PET economy. Genes.

[73] Trifunović D., Schuchmann K., Müller V. (2016). Ethylene glycol metabolism in the acetogen *Acetobacterium woodii*. J. Bacteriol..

[74] Mückschel B., Simon O., Klebensberger J., Graf N., Rosche B., Altenbuchner J., Pfannstiel J., Huber A., Hauer B. (2012). Ethylene glycol metabolism by Pseudomonas putida. Appl. Environ. Microbiol..

[75] Franden M.A., Jayakody L.N., Li W.-J., Wagner N.J., Cleveland N.S., Michener W.E., Hauer B., Blank L.M., Wierckx N., Klebensberger J. (2018). Engineering Pseudomonas putida KT2440 for efficient ethylene glycol utilization. Metab. Eng..

[76] Kumar R.V., Kanna G.R., Elumalai S. (2017). Biodegradation of polyethylene by green photosynthetic microalgae. J. Bioremediat. Biodegrad..

[77] Zheng Y., Yanful E.K., Bassi A.S. (2005). A review of plastic waste biodegradation. Crit. Rev. Biotechnol..

[78] Krueger M.C., Harms H., Schlosser D. (2015). Prospects for microbiological solutions to environmental pollution with plastics. Appl. Microbiol. Biotechnol..

[79] Nishida H., Tokiwa Y. (1993). Distribution of poly (β-hydroxybutyrate) and poly (ε-caprolactone) aerobic degrading microorganisms in different environments. J. Environ. Polym. Degrad..

[80] Parikh M., Gross R. (1992). The effect of crystalline morphology on enzymatic degradation kinetics. Polym. Mater. Sci. Eng. (PMSE).

[81] Marten E., Müller R.-J., Deckwer W.-D. (2003). Studies on the enzymatic hydrolysis of polyesters I. Low molecular mass model esters and aliphatic polyesters. Polym. Degrad. Stab..

[82] Marten E., Müller R.-J., Deckwer W.-D. (2005). Studies on the enzymatic hydrolysis of polyesters. II. Aliphatic–aromatic copolyesters. Polym. Degrad. Stab..

[83] Tokiwa Y., Calabia B.P., Ugwu C.U., Aiba S. (2009). Biodegradability of plastics. Int. J. Mol. Sci..

[84] Wei R., Zimmermann W. (2017). Microbial enzymes for the recycling of recalcitrant petroleum-based plastics: How far are we?. Microb. Biotechnol..

[85] Vertommen M., Nierstrasz V., Van Der Veer M., Warmoeskerken M. (2005). Enzymatic surface modification of poly (ethylene terephthalate). J. Biotechnol..

[86] Müller R.J., Schrader H., Profe J., Dresler K., Deckwer W.D. (2005). Enzymatic degradation of poly (ethylene terephthalate): Rapid hydrolyse using a hydrolase from *T. fusca*. Macromol. Rapid Commun..

[87] Mohanan N., Montazer Z., Sharma P.K., Levin D.B. (2020). Microbial and Enzymatic Degradation of Synthetic Plastics. Front. Microbiol..

[88] Austin H.P., Allen M.D., Donohoe B.S., Rorrer N.A., Kearns F.L., Silveira R.L., Pollard B.C., Dominick G., Duman R., El Omari K. (2018). Characterization and engineering of a plastic-degrading aromatic polyesterase. Proc. Natl. Acad. Sci. USA.

[89] Wei R., Oeser T., Then J., Kühn N., Barth M., Schmidt J., Zimmermann W. (2014). Functional characterization and structural modeling of synthetic polyester-degrading hydrolases from Thermomonospora curvata. AMB Express.

[90] Remington S., Franken S., Sussman J., Frolow F., Ollis D., Verschueren K., Cheah E., Cygler M., Dijkstra B., Harel M. (1992). The alpha/beta hydrolase fold. Protein Eng..

[91] Barth M., Oeser T., Wei R., Then J., Schmidt J., Zimmermann W. (2015). Effect of hydrolysis products on the enzymatic degradation of polyethylene terephthalate nanoparticles by a polyester hydrolase from Thermobifida fusca. Biochem. Eng. J..

[92] Sulaiman S., Yamato S., Kanaya E., Kim J.-J., Koga Y., Takano K., Kanaya S. (2012). Isolation of a novel cutinase homolog with polyethylene terephthalate-degrading activity from leaf-branch compost by using a metagenomic approach. Appl. Environ. Microbiol..

[93] Kawabata T., Oda M., Kawai F. (2017). Mutational analysis of cutinase-like enzyme, Cut190, based on the 3D docking structure with model compounds of polyethylene terephthalate. J. Biosci. Bioeng..

[94] Tournier V., Topham C., Gilles A., David B., Folgoas C., Moya-Leclair E., Kamionka E., Desrousseaux M.-L., Texier H., Gavalda S. (2020). An engineered PET depolymerase to break down and recycle plastic bottles. Nature.

[95] Kim H.R., Song W.S. (2006). Lipase treatment of polyester fabrics. Fibers Polym..

[96] Guebitz G.M., Cavaco-Paulo A. (2008). Enzymes go big: Surface hydrolysis and functionalisation of synthetic polymers. Trends Biotechnol..

[97] Ribitsch D., Herrero Acero E., Greimel K., Dellacher A., Zitzenbacher S., Marold A., Rodriguez R.D., Steinkellner G., Gruber K., Schwab H. (2012). A new esterase from Thermobifida halotolerans hydrolyses polyethylene terephthalate (PET) and polylactic acid (PLA). Polymers.

[98] Sales J.C.S., de Castro A.M., Ribeiro B.D., Coelho M.A.Z. (2020). Supplementation of watermelon peels as an enhancer of lipase and esterase production by Yarrowia lipolytica in solid-state fermentation and their potential use as biocatalysts in poly (ethylene terephthalate)(PET) depolymerization reactions. Biocatal. Biotransform..

[99] Roth C., Wei R., Oeser T., Then J., Föllner C., Zimmermann W., Sträter N. (2014). Structural and functional studies on a thermostable polyethylene terephthalate degrading hydrolase from Thermobifida fusca. Appl. Microbiol. Biotechnol..

[100] Sulaiman S., You D.-J., Kanaya E., Koga Y., Kanaya S. (2014). Crystal structure and thermodynamic and kinetic stability of metagenome-derived LC-cutinase. Biochemistry.

[101] Then J., Wei R., Oeser T., Barth M., Belisário-Ferrari M.R., Schmidt J., Zimmermann W. (2015). Ca2+ and Mg2+ binding site engineering increases the degradation of polyethylene terephthalate films by polyester hydrolases from Thermobifida fusca. Biotechnol. J..

[102] Thumarat U., Nakamura R., Kawabata T., Suzuki H., Kawai F. (2012). Biochemical and genetic analysis of a cutinase-type polyesterase from a thermophilic Thermobifida alba AHK119. Appl. Microbiol. Biotechnol..

[103] Herrero Acero E., Ribitsch D., Steinkellner G., Gruber K., Greimel K., Eiteljoerg I., Trotscha E., Wei R., Zimmermann W., Zinn M. (2011). Enzymatic surface hydrolysis of PET: Effect of structural diversity on kinetic properties of cutinases from Thermobifida. Macromolecules.

[104] Kawai F., Oda M., Tamashiro T., Waku T., Tanaka N., Yamamoto M., Mizushima H., Miyakawa T., Tanokura M. (2014). A novel Ca2+-activated, thermostabilized polyesterase capable of hydrolyzing polyethylene terephthalate from *Saccharomonospora viridis* AHK190. Appl. Microbiol. Biotechnol..

[105] Purdy R., Kolattukudy P. (1975). Hydrolysis of plant cuticle by plant pathogens. Purification, amino acid composition, and molecular weight of two isoenzymes of cutinase and a nonspecific esterase from *Fusarium solani* f. *pisi*. Biochemistry.

[106] Furukawa M., Kawakami N., Tomizawa A., Miyamoto K. (2019). Efficient degradation of poly (ethylene terephthalate) with Thermobifida fusca cutinase exhibiting improved catalytic activity generated using mutagenesis and additive-based approaches. Sci. Rep..

[107] Ronkvist Å.M., Xie W., Lu W., Gross R.A. (2009). Cutinase-Catalyzed Hydrolysis of Poly(ethylene terephthalate). Macromolecules.

[108] Zimmermann W., Billig S. (2010). Enzymes for the biofunctionalization of poly (ethylene terephthalate). Biofunctionalization of Polymers and Their Applications.

[109] Eberl A., Heumann S., Brückner T., Araujo R., Cavaco-Paulo A., Kaufmann F., Kroutil W., Guebitz G.M. (2009). Enzymatic surface hydrolysis of poly (ethylene terephthalate) and bis (benzoyloxyethyl) terephthalate by lipase and cutinase in the presence of surface active molecules. J. Biotechnol..

[110] Ribitsch D., Heumann S., Trotscha E., Herrero Acero E., Greimel K., Leber R., Birner-Gruenberger R., Deller S., Eiteljoerg I., Remler P. (2011). Hydrolysis of polyethyleneterephthalate by p-nitrobenzylesterase from Bacillus subtilis. Biotechnol. Prog..

[111] Kleeberg I., Hetz C., Kroppenstedt R.M., Müller R.-J., Deckwer W.-D. (1998). Biodegradation of aliphatic-aromatic copolyesters by Thermomonospora fusca and other thermophilic compost isolates. Appl. Environ. Microbiol..

[112] Kleeberg I., Welzel K., VandenHeuvel J., Müller R.-J., Deckwer W.-D. (2005). Characterization of a New Extracellular Hydrolase from Thermobifida fusca Degrading Aliphatic−Aromatic Copolyesters. Biomacromolecules.

[113] Chen S., Su L., Billig S., Zimmermann W., Chen J., Wu J. (2010). Biochemical characterization of the cutinases from *Thermobifida fusca*. J. Mol. Catal. B Enzym..

[114] Oda M., Yamagami Y., Inaba S., Oida T., Yamamoto M., Kitajima S., Kawai F. (2018). Enzymatic hydrolysis of PET: Functional roles of three Ca 2+ ions bound to a cutinase-like enzyme, Cut190*, and its engineering for improved activity. Appl. Microbiol. Biotechnol..

[115] Cruz H., Pérez C., Wellington E., Castro C., Servín-González L. (1994). Sequence of the *Streptomyces albus* G lipase-encoding gene reveals the presence of a prokaryotic lipase family. Gene.

[116] Pérez C., Juárez K., García-Castells E., Soberón G., Servín-González L. (1993). Cloning, characterization, and expression in Streptomyces lividans 66 of an extracellular lipase-encoding gene from *Streptomyces* sp. M11. Gene.

[117] Lykidis A., Mavromatis K., Ivanova N., Anderson I., Land M., DiBartolo G., Martinez M., Lapidus A., Lucas S., Copeland A. (2007). Genome sequence and analysis of the soil cellulolytic actinomycete *Thermobifida fusca* YX. J. Bacteriol..

[118] Chen V.B., Arendall W.B., Headd J.J., Keedy D.A., Immormino R.M., Kapral G.J., Murray L.W., Richardson J.S., Richardson D.C. (2010). MolProbity: All-atom structure validation for macromolecular crystallography. Acta Crystallogr. Sect. D Biol. Crystallogr..

[119] Alisch-Mark M., Herrmann A., Zimmermann W. (2006). Increase of the hydrophilicity of polyethylene terephthalate fibres by hydrolases from *Thermomonospora fusca* and *Fusarium solani* f. sp. *pisi*. Biotechnol. Lett..

[120] Feuerhack A., Alisch-Mark M., Kisner A., Pezzin S., Zimmermann W., Andreaus J. (2008). Biocatalytic surface modification of knitted fabrics made of poly (ethylene terephthalate) with hydrolytic enzymes from *Thermobifida fusca* KW3b. Biocatal. Biotransform..

[121] Hegde K., Veeranki V.D. (2013). Production optimization and characterization of recombinant cutinases from *Thermobifida fusca* sp. NRRL B-8184. Appl. Biochem. Biotechnol..

[122] Chertkov O., Sikorski J., Nolan M., Lapidus A., Lucas S., Del Rio T.G., Tice H., Cheng J.-F., Goodwin L., Pitluck S. (2011). Complete genome sequence of *Thermomonospora curvata* type strain (B9). Stand. Genom. Sci..

[123] Arima J., Uesugi Y., Uraji M., Yatsushiro S., Tsuboi S., Iwabuchi M., Hatanaka T. (2006). Modulation of *Streptomyces leucine* aminopeptidase by calcium: Identification and functional analysis of key residues in activation and stabilization by calcium. J. Biol. Chem..

[124] Almeida E.L., Carrillo Rincón A.F., Jackson S.A., Dobson A.D.W. (2019). In silico Screening and Heterologous Expression of a Polyethylene Terephthalate Hydrolase (PETase)-Like Enzyme (SM14est) with Polycaprolactone (PCL)-Degrading Activity, From the Marine Sponge-Derived Strain *Streptomyces* sp. SM14. Front. Microbiol..

[125] Jabloune R., Khalil M., Ben Moussa I.E., Simao-Beaunoir A.-M., Lerat S., Brzezinski R., Beaulieu C. (2020). Enzymatic Degradation of p-Nitrophenyl Esters, Polyethylene Terephthalate, Cutin, and Suberin by Sub1, a Suberinase Encoded by the Plant Pathogen *Streptomyces scabies*. Microbes Environ..

[126] Alves N., Mano J.F., Balaguer E., Dueñas J.M., Ribelles J.G. (2002). Glass transition and structural relaxation in semi-crystalline poly (ethylene terephthalate): A DSC study. Polymer.

[127] Kikkawa Y., Fujita M., Abe H., Doi Y. (2004). Effect of water on the surface molecular mobility of poly (lactide) thin films: An atomic force microscopy study. Biomacromolecules.

[128] Taniguchi I., Yoshida S., Hiraga K., Miyamoto K., Kimura Y., Oda K. (2019). Biodegradation of PET: Current status and application aspects. ACS Catal..

[129] Dimarogona M., Nikolaivits E., Kanelli M., Christakopoulos P., Sandgren M., Topakas E. (2015). Structural and functional studies of a Fusarium oxysporum cutinase with polyethylene terephthalate modification potential. Biochim. Biophys. Acta (BBA) Gen. Subj..

[130] Gamerith C., Vastano M., Ghorbanpour S.M., Zitzenbacher S., Ribitsch D., Zumstein M.T., Sander M., Herrero Acero E., Pellis A., Guebitz G.M. (2017). Enzymatic degradation of aromatic and aliphatic polyesters by P. pastoris expressed cutinase 1 from *Thermobifida cellulosilytica*. Front. Microbiol..

[131] Tanasupawat S., Takehana T., Yoshida S., Hiraga K., Oda K. (2016). *Ideonella sakaiensis* sp. nov., isolated from a microbial consortium that degrades poly (ethylene terephthalate). Int. J. Syst. Evol. Microbiol..

[132] Uchida H., Shigeno-Akutsu Y., Nomura N., Nakahara T., Nakajima-Kambe T. (2002). Cloning and sequence analysis of poly (tetramethylene succinate) depolymerase from *Acidovorax delafieldii* strain BS-3. J. Biosci. Bioeng..

[133] Tang B., Yu Y., Zhang Y., Zhao G., Ding X. (2015). Complete genome sequence of the glidobactin producing strain [Polyangium] brachysporum DSM 7029. J. Biotechnol..

[134] Anantharaman K., Brown C.T., Hug L.A., Sharon I., Castelle C.J., Probst A.J., Thomas B.C., Singh A., Wilkins M.J., Karaoz U. (2016). Thousands of microbial genomes shed light on interconnected biogeochemical processes in an aquifer system. Nat. Commun..

[135] Silva C.M., Carneiro F., O’Neill A., Fonseca L.P., Cabral J.S., Guebitz G., Cavaco-Paulo A. (2005). Cutinase—A new tool for biomodification of synthetic fibers. J. Polym. Sci. Part A Polym. Chem..

[136] Sasoh M., Masai E., Ishibashi S., Hara H., Kamimura N., Miyauchi K., Fukuda M. (2006). Characterization of the terephthalate degradation genes of *Comamonas* sp. strain E6. Appl. Environ. Microbiol..

[137] Hosaka M., Kamimura N., Toribami S., Mori K., Kasai D., Fukuda M., Masai E. (2013). Novel tripartite aromatic acid transporter essential for terephthalate uptake in *Comamonas* sp. strain E6. Appl. Environ. Microbiol..

[138] Pérez-Pantoja D., Donoso R., Agulló L., Córdova M., Seeger M., Pieper D.H., González B. (2012). Genomic analysis of the potential for aromatic compounds biodegradation in Burkholderiales. Environ. Microbiol..

[139] Wilkes R.A., Aristilde L. (2017). Degradation and metabolism of synthetic plastics and associated products by *Pseudomonas* sp.: Capabilities and challenges. J. Appl. Microbiol..

[140] Li W.J., Jayakody L.N., Franden M.A., Wehrmann M., Daun T., Hauer B., Blank L.M., Beckham G.T., Klebensberger J., Wierckx N. (2019). Laboratory evolution reveals the metabolic and regulatory basis of ethylene glycol metabolism by *Pseudomonas putida* KT2440. Environ. Microbiol..

[141] Tiwari V. (2016). In vitro engineering of novel bioactivity in the natural enzymes. Front. Chem..

[142] Fecker T., Galaz-Davison P., Engelberger F., Narui Y., Sotomayor M., Parra L.P., Ramírez-Sarmiento C.A. (2018). Active site flexibility as a hallmark for efficient PET degradation by I. sakaiensis PETase. Biophys. J..

[143] Liu B., He L., Wang L., Li T., Li C., Liu H., Luo Y., Bao R. (2018). Protein crystallography and site-direct mutagenesis analysis of the poly (ethylene terephthalate) hydrolase PETase from *Ideonella sakaiensis*. ChemBioChem.

[144] Joo S., Cho I.J., Seo H., Son H.F., Sagong H.-Y., Shin T.J., Choi S.Y., Lee S.Y., Kim K.-J. (2018). Structural insight into molecular mechanism of poly (ethylene terephthalate) degradation. Nat. Commun..

[145] Chen S., Tong X., Woodard R.W., Du G., Wu J., Chen J. (2008). Identification and characterization of bacterial cutinase. J. Biol. Chem..

[146] Palm G.J., Reisky L., Böttcher D., Müller H., Michels E.A., Walczak M.C., Berndt L., Weiss M.S., Bornscheuer U.T., Weber G. (2019). Structure of the plastic-degrading Ideonella sakaiensis MHETase bound to a substrate. Nat. Commun..

[147] Matak M.Y., Moghaddam M.E. (2009). The role of short-range Cys171–Cys178 disulfide bond in maintaining cutinase active site integrity: A molecular dynamics simulation. Biochem. Biophys. Res. Commun..

[148] Egmond M.R., De Vlieg J. (2000). *Fusarium solani* pisi cutinase. Biochimie.

[149] Cunha M.T., Costa M.J., Calado C.R., Fonseca L.P., Aires-Barros M.R., Cabral J.M. (2003). Integration of production and aqueous two-phase systems extraction of extracellular *Fusarium solani* pisi cutinase fusion proteins. J. Biotechnol..

[150] Longhi S., Cambillau C. (1999). Structure-activity of cutinase, a small lipolytic enzyme. Biochim. Biophys. Acta.

[151] Gindro K., Pezet R. (1999). Purification and characterization of a 40.8-kDa cutinase in ungerminated conidia of *Botrytis cinerea* Pers.: Fr. FEMS Microbiol. Lett..

[152] Chen Z., Franco C.F., Baptista R.P., Cabral J.M.S., Coelho A.V., Rodrigues C.J., Melo E.P. (2007). Purification and identification of cutinases from *Colletotrichum kahawae* and *Colletotrichum gloeosporioides*. Appl. Microbiol. Biotechnol..

[153] Wang G.Y., Michailides T.J., Hammock B.D., Lee Y.M., Bostock R.M. (2002). Molecular cloning, characterization, and expression of a redox-responsive cutinase from *Monilinia fructicola* (Wint.) Honey. Fungal Genet. Biol..

[154] Wang G.Y., Michailides T.J., Hammock B.D., Lee Y.M., Bostock R.M. (2000). Affinity purification and characterization of a cutinase from the fungal plant pathogen *Monilinia fructicola* (Wint.) honey. Arch. Biochem. Biophys..

[155] Sweigard J.A., Carroll A.M., Farrall L., Chumley F.G., Valent B. (1998). Magnaporthe grisea pathogenicity genes obtained through insertional mutagenesis. Mol. Plant Microbe Interact..

[156] Maeda H., Yamagata Y., Abe K., Hasegawa F., Machida M., Ishioka R., Gomi K., Nakajima T. (2005). Purification and characterization of a biodegradable plastic-degrading enzyme from *Aspergillus oryzae*. Appl. Microbiol. Biotechnol..

[157] Xu H., Yan Q., Duan X., Yang S., Jiang Z. (2015). Characterization of an acidic cold-adapted cutinase from Thielavia terrestris and its application in flavor ester synthesis. Food Chem..

[158] Yang S., Xu H., Yan Q., Liu Y., Zhou P., Jiang Z. (2013). A low molecular mass cutinase of *Thielavia terrestris* efficiently hydrolyzes poly(esters). J. Ind. Microbiol. Biotechnol..

[159] Rogers L.M., Flaishman M.A., Kolattukudy P.E. (1994). Cutinase gene disruption in *Fusarium solani* f sp pisi decreases its virulence on pea. Plant Cell.

[160] Li D., Ashby A.M., Johnstone K. (2003). Molecular Evidence that the Extracellular Cutinase Pbc1 Is Required for Pathogenicity of *Pyrenopeziza brassicae* on Oilseed Rape. Mol. Plant-Microbe Interact..

[161] Liu T., Hou J., Wang Y., Jin Y., Borth W., Zhao F., Liu Z., Hu J., Zuo Y. (2016). Genome-wide identification, classification and expression analysis in fungal–plant interactions of cutinase gene family and functional analysis of a putative ClCUT7 in *Curvularia lunata*. Mol. Genet. Genom..

[162] Wang Y., Chen J., Li D.-W., Zheng L., Huang J. (2017). CglCUT1 gene required for cutinase activity and pathogenicity of *Colletotrichum gloeosporioides* causing anthracnose of *Camellia oleifera*. Eur. J. Plant Pathol..

[163] Sweigard J.A., Chumley F.G., Valent B. (1992). Disruption of a *Magnaporthe grisea* cutinase gene. Mol. Gen. Genet..

[164] Skamnioti P., Gurr S.J. (2007). Magnaporthe grisea Cutinase2 Mediates Appressorium Differentiation and Host Penetration and Is Required for Full Virulence. Plant Cell.

[165] Wilson R.A., Talbot N.J. (2009). Under pressure: Investigating the biology of plant infection by *Magnaporthe oryzae*. Nat. Rev. Microbiol..

[166] Martinez C., De Geus P., Lauwereys M., Matthyssens G., Cambillau C. (1992). *Fusarium solani* cutinase is a lipolytic enzyme with a catalytic serine accessible to solvent. Nature.

[167] Nikolaivits E., Kanelli M., Dimarogona M., Topakas E. (2018). A Middle-Aged Enzyme Still in Its Prime: Recent Advances in the Field of Cutinases. Catalysts.

[168] Nishida H., Tokiwa Y. (1993). Effects of higher-order structure of poly (3-hydroxybutyrate) on its biodegradation. II. Effects of crystal structure on microbial degradation. J. Environ. Polym. Degrad..

[169] Abe H., Doi Y. (1999). Structural effects on enzymatic degradabilities for poly[(R)-3-hydroxybutyric acid] and its copolymers. Int. J. Biol. Macromol..

[170] Seretoudi G., Bikiaris D.N., Panayiotou C. (2002). Synthesis, characterization and biodegradability of poly(ethylene succinate)/poly+µ-caprolactone) block copolymers. Polymer.

[171] Pudack C., Stepanski M., Fässler P. (2020). PET Recycling—Contributions of Crystallization to Sustainability. Chem. Ing. Tech..

[172] Mateo C., Palomo J.M., Fernandez-Lorente G., Guisan J.M., Fernandez-Lafuente R. (2007). Improvement of enzyme activity, stability and selectivity via immobilization techniques. Enzym. Microb. Technol..

[173] Kumari V., Kumar S., Kaur I., Bhalla T.C. (2017). Graft copolymerization of acrylamide on chitosan-co-chitin and its application for immobilization of *Aspergillus* sp. RL2Ct cutinase. Bioorganic Chem..

[174] Sousa I.T., Lourenço N.M.T., Afonso C.A.M., Taipa M.A. (2013). Protein stabilization with a dipeptide-mimic triazine-scaffolded synthetic affinity ligand. J. Mol. Recognit..

[175] Wang Z., Su T., Zhao J. (2021). Immobilization of Fusarium solani Cutinase onto Magnetic Genipin-Crosslinked Chitosan Beads. Catalysts.

[176] Zhang C., Zeng G., Huang D., Lai C., Huang C., Li N., Xu P., Cheng M., Zhou Y., Tang W. (2014). Combined removal of di (2-ethylhexyl) phthalate (DEHP) and Pb (II) by using a cutinase loaded nanoporous gold-polyethyleneimine adsorbent. Rsc Adv..

[177] Obregón W.D., Cisneros J.S., Ceccacci F., Quiroga E. (2015). A highly stable biocatalyst obtained from covalent immobilization of a non-commercial cysteine phytoprotease. J. Bioprocess. Biotech..

[178] Gupta M. (1991). Thermostabilization of proteins. Biotechnol. Appl. Biochem. (USA).

[179] Ma M.M., Wang L.Y., Zhu H.Y. (2012). Enzymatic degradation of polyester-nanoparticles by lipases and adsorption of lipases on the polyester-nanoparticles. Advanced Materials Research.

[180] Wang X., Lu D., Jönsson L.J., Hong F. (2008). Preparation of a PET-Hydrolyzing Lipase from *Aspergillus oryzae* by the Addition of Bis(2-hydroxyethyl) Terephthalate to the Culture Medium and Enzymatic Modification of PET Fabrics. Eng. Life Sci..

[181] Carniel A., Valoni É., Nicomedes J., Gomes A.d.C., Castro A.M.d. (2017). Lipase from *Candida antarctica* (CALB) and cutinase from *Humicola insolens* act synergistically for PET hydrolysis to terephthalic acid. Process Biochem..

[182] de Castro A.M., Carniel A., Nicomedes Junior J., da Conceição Gomes A., Valoni É. (2017). Screening of commercial enzymes for poly(ethylene terephthalate) (PET) hydrolysis and synergy studies on different substrate sources. J. Ind. Microbiol. Biotechnol..

[183] Henderson B., Curtis M., Seymour R., Donos N. (2009). Periodontal Medicine and Systems Biology.

[184] Garza D.R., Dutilh B.E. (2015). From cultured to uncultured genome sequences: Metagenomics and modeling microbial ecosystems. Cell. Mol. Life Sci..

[185] Parages M.L., Gutiérrez-Barranquero J.A., Reen F.J., Dobson A.D., O’Gara F. (2016). Integrated (Meta) Genomic and Synthetic Biology Approaches to Develop New Biocatalysts. Mar. Drugs.

[186] Danso D., Schmeisser C., Chow J., Zimmermann W., Wei R., Leggewie C., Li X., Hazen T., Streit W.R., Parales R.E. (2018). New Insights into the Function and Global Distribution of Polyethylene Terephthalate (PET)-Degrading Bacteria and Enzymes in Marine and Terrestrial Metagenomes. Appl. Environ. Microbiol..

[187] Meilleur C., Hupé J.-F., Juteau P., Shareck F. (2009). Isolation and characterization of a new alkali-thermostable lipase cloned from a metagenomic library. J. Ind. Microbiol. Biotechnol..

[188] Baumann P., Baumann L., Bang S.S., Woolkalis M.J. (1980). Reevaluation of the taxonomy ofVibrio, beneckea, and Photobacterium: Abolition of the genus Beneckea. Curr. Microbiol..

[189] Dodd D., Mackie R.I., Cann I.K.O. (2011). Xylan degradation, a metabolic property shared by rumen and human colonic Bacteroidetes. Mol. Microbiol..

[190] Thomas F., Hehemann J.-H., Rebuffet E., Czjzek M., Michel G. (2011). Environmental and Gut Bacteroidetes: The Food Connection. Front. Microbiol..

[191] Foley M.H., Cockburn D.W., Koropatkin N.M. (2016). The Sus operon: A model system for starch uptake by the human gut Bacteroidetes. Cell. Mol. Life Sci..

[192] Zhou D., Chen J., Wu J., Yang J., Wang H. (2021). Biodegradation and catalytic-chemical degradation strategies to mitigate microplastic pollution. Sustain. Mater. Technol..

[193] Furukawa M., Kawakami N., Oda K., Miyamoto K. (2018). Acceleration of enzymatic degradation of poly (ethylene terephthalate) by surface coating with anionic surfactants. ChemSusChem.

